# Best practices in the African Medicines Regulatory Harmonization initiative: Perspectives of regulators and medicines manufacturers

**DOI:** 10.1371/journal.pgph.0001651

**Published:** 2023-04-26

**Authors:** Margareth Ndomondo-Sigonda, Samvel Azatyan, Petra Doerr, Collins Agaba, Kristin N. Harper

**Affiliations:** 1 African Union Development Agency–New Partnership for Africa’s Development, Midrand, South Africa; 2 Regulatory Convergence and Networks, World Health Organization, Geneva, Switzerland; 3 European Directorate for the Quality of Medicines & Healthcare, Strasbourg, France; 4 CORE Consulting LTD, Mbarara, Uganda; 5 Harper Health & Science Communications, LLC, Seattle, Washington, United States of America; Universidade de São Paulo: Universidade de Sao Paulo, BRAZIL

## Abstract

In the African Medicines Regulatory Harmonization initiative, national regulatory authorities (NRAs) within each of Africa’s regional economic communities coordinate their activities, rely on the work of one another and other trusted regulatory authorities, and apply other principles of smart regulation. The first regional medicines regulatory harmonization (MRH) initiative in Africa was launched in 2012, with the goal of accelerating access to quality, safe, effective medical products, and now five MRH initiatives are active on the continent. Thus, a wealth of knowledge regarding best practices and approaches to dealing with common challenges has accumulated. The goal of this qualitative study was to gather and share information on these best practices. To do this, we conducted interviews with key participants from four regional MRH initiatives—the East African Community (EAC), Southern African Development Community (SADC), Economic Community of West African States (ECOWAS), and Intergovernmental Authority on Development (IGAD)—as well as representatives from the pharmaceutical industry. Here we explore major themes that emerged from the interviews: 1. Transparency and reliability are critical; 2. Reliance is essential for smart regulation; 3. Multiple successful strategies for NRA capacity building have been identified; 4. Communication between heads of agencies is essential; 5. Cooperation at the regional level is not possible without leadership at the NRA level; 6. Sustainable funding remains challenging; and 7. Industry has important insights. We hope that the information on best practices shared in this article can benefit regional MRH initiatives inside and outside of Africa, ultimately helping them accelerate access to quality, safe, effective medical products.

## Introduction

The African Medicines Regulatory Harmonization (AMRH) initiative was launched to accelerate access to quality, safe, effective medical products by optimizing the regulatory environment on the continent [1]. In particular, it was meant to address the significant delays in marketing authorization timelines for important medicines that many African countries experience [1–3]. Marketing authorization is an official document issued by a regulatory authority that allows a product to be marketed or distributed after it has been evaluated for safety, efficacy, and quality [4]. The marketing authorization timeline is the period between when a medicines manufacturer submits a marketing authorization application and when a regulatory authority issues its decision. In some areas of the world, medicines manufacturers can submit a single marketing authorization application to an organization that has the ability to grant marketing authorization for a large number of countries. For example, a manufacturer that submits an application to the European Medicines Agency (EMA) can obtain marketing authorization for a large number of European countries. However, it is more typical for a manufacturer to have to submit individual applications to the national regulatory authority (NRA) for each country they would like to market or distribute their product in. This process, which often involves preparing an extensive application specific to the country, paying a fee to the country’s NRA, and engaging in back and forth with the NRA to answer questions, may take years. In addition, manufacturers may have little incentive to submit applications to countries that they feel do not represent lucrative markets. As a result, after a novel medicine is approved for the first time, usually in a high-income country such as the United States, it takes an average of 4 to 7 years for that medicine to be approved in sub-Saharan African countries [2].

The AMRH initiative’s goal was for NRAs within each of Africa’s regional economic communities (RECs) to address this problem by coordinating their activities, relying on the work of one another and other trusted regulatory authorities, and applying other principles of smart regulation [5]. Specifically, NRAs within an REC would harmonize technical requirements and standards for medical products regulation, perform joint reviews of marketing authorization applications and joint inspections of manufacturing sites, and increase the use of reliance and cooperation in regulatory matters related to medical products [3, 6–9]. The resulting improvements in regulatory efficiency would make it easier and faster for medicines manufacturers to register quality products [3, 10]. However, this level of reliance and cooperation requires trust between NRAs, as well as a minimum level of regulatory maturity in each partner country. Therefore, regulatory harmonization also requires capacity building in countries with less mature regulatory systems. To aid in this process, the World Health Organization (WHO) offers the Global Benchmarking Tool, a global standard for assessing each NRA’s regulatory capacity in various areas [11, 12]. NRAs can opt for either self-benchmarking, assisted by WHO staff, or formal benchmarking, performed by an evaluator from WHO. In addition to assigning each NRA an overall maturity level, the Global Benchmarking Tool allows it to identify its specific strengths and weaknesses. WHO then works with NRAs to create and carry out institutional development plans that strengthen their regulatory systems.

Today, five African RECs have taken up the challenge of creating medicines regulatory harmonization (MRH) initiatives: the East African Community (EAC), Southern African Development Community (SADC), Economic Community of West African States (ECOWAS), Intergovernmental Authority on Development (IGAD), and Economic Community of Central African States (ECCAS). The AMRH initiative thus represents a natural experiment in which each REC has developed its own unique approach to MRH. The goal of this study was to gather and share information on the best practices unlocked by the various regional MRH initiatives.

Although the first regional MRH was launched in 2012, the history of the AMRH initiative goes back further [3]. The need for harmonization and cooperation in medicines regulation had long been recognized in Africa. This need was formalized in the 2007 African Union’s Pharmaceutical Manufacturing Plan for Africa, a critical component of which was to provide an enabling environment for the development of the pharmaceutical industry. In 2009, a consortium of partners, including NRAs, RECs, the African Union Development Agency–New Partnership for Africa’s Development (AUDA-NEPAD), the Pan African Parliament, WHO, the Bill & Melinda Gates Foundation (BMGF), the UK’s Foreign, Commonwealth and Development Office (FCDO), and the Clinton Health Access Initiative came together to address this need by establishing the AMRH initiative. In 2011, AUDA-NEPAD created a comprehensive 5-year strategic plan for the AMRH initiative, and the Global Medicines Regulatory Harmonization Multi-Donor Trust Fund was created. This trust fund, managed by the World Bank, was meant to jumpstart the regional MRH initiatives. The first regional MRH initiative, that of the EAC, began in 2012; the most recent regional MRH initiative to come online was that of IGAD, which was endorsed by the heads of its NRAs in 2017.

Regional MRH initiatives are complex undertakings. Each one involves many players: the countries in a given REC; donors such as BMGF, FCDO, the US Government, and the Swiss Agency for Development and Cooperation; technical implementation partners, such as WHO and Swissmedic; AUDA-NEPAD, which coordinates and advocates for the initiatives; and the World Bank, which manages the trust fund. Each initiative must also perform many different activities simultaneously, including harmonizing guidelines and procedures, work sharing (i.e., carrying out joint assessments and inspections), training and capacity building, and pharmacovigilance and post-marketing surveillance in some cases. Finally, each initiative must deal with multiple challenges, including coordinating member countries with different levels of regulatory capacity, taking leadership of the initiatives at the NRA level, sharing information efficiently between countries, and developing and securing political support for the legal framework required. Other hurdles include convincing industry to participate in regional assessments and inspections; leveraging regional assessment outputs for greater regulatory efficiency and to issue national marketing authorizations; dealing with the decisions of some companies not to offer their products in certain markets for a variety of reasons; and developing adequate, sustainable sources of funding.

**Fig 1:**
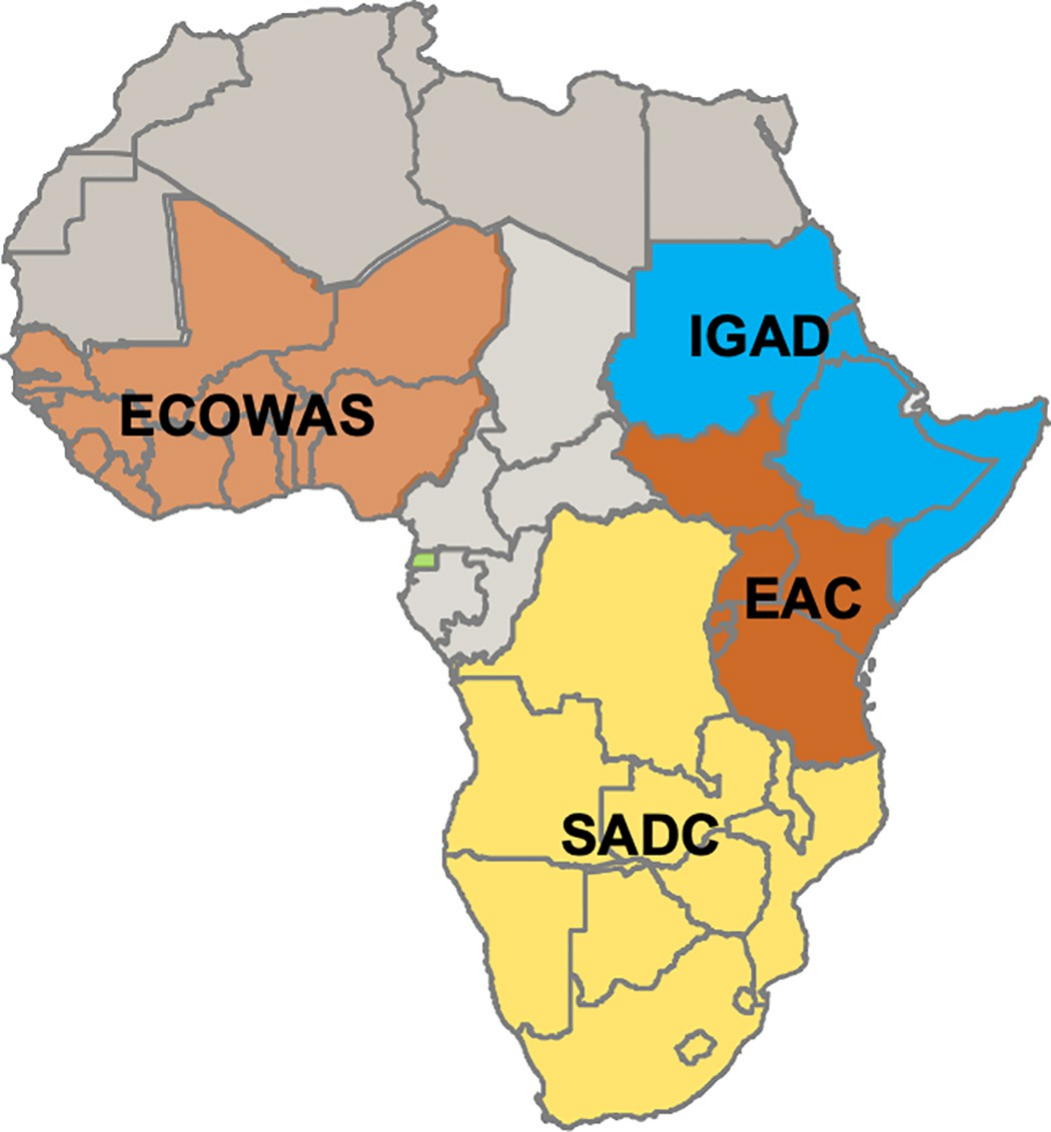
The four medicines regulatory harmonization initiatives featured in this study are coordinated by the four corresponding regional economic communities pictured here: The East African Community (EAC), Southern African Development Community (SADC), Economic Community of West African States (ECOWAS), and Intergovernmental Authority on Development (IGAD). Adapted from a map published in Ndomondo-Sigonda et al, 2020 [10] and modified and published here under the CC-BY 4.0 license.

Given the complexity of the challenges that the regional MRH initiatives face, it is important to identify effective strategies as they arise, so that they can be disseminated. Research shows that without studies specifically designed to uncover best practices within an institution, such as the AMRH initiative, good ideas and stellar performance often go unnoticed, preventing other sites or institutions from learning about and adopting advances [13]. To ensure that best practices developed within the AMRH initiative were identified and described, we conducted interviews with key participants from four regional MRH initiatives—EAC, SADC, ECOWAS, and IGAD (Fig 1)—as well as representatives from the pharmaceutical industry. Here, we share the results of a qualitative analysis of these interviews, in hopes that the best practices that emerge could benefit regional MRH initiatives.

## Methods

### Study design and participants

This was a qualitative study of regional MRH initiatives aimed at gaining an in-depth understanding of best practices. From October 2020 to January 2021, interviews were conducted with 12 key informants from four regional MRH initiatives (EAC, SADC, ECOWAS, and IGAD). For each regional MRH initiative, two heads of NRAs and one member of the Secretariat were interviewed by the same interviewer (K.H.), who had no involvement in the regional MRH initiatives, including in their planning, implementation, or funding. In addition, four industry representatives were interviewed, also by K.H. One was from an Africa-based generics company, one was a regulatory affairs expert at an international trade association, and two were from multinational pharmaceutical companies.

### Theoretical framework

Here, we define best practices as those that are perceived to demonstrate effectiveness in the context of program objectives, and which have the potential to be adopted, with similarly positive outcomes, in other settings [14]. We used the RE-AIM (Reach, Effectiveness, Adoption, Implementation, and Maintenance) model to inform our interview questions [15]. Specifically, after identifying best practice themes from respondents’ interviews, we also used their responses to assess how effective a given practice was perceived to be, how many regional initiatives implemented that practice, how diligently the practice was implemented by those initiatives, and whether the practice was maintained over time.

### Data collection methods

Author K.H. conducted the interviews. She has a Master’s degree in public health and experience working with regulators on the African continent, as well as with conducting interviews and qualitative research. All interviews followed a semi-structured format, consisting of open-ended, pre-specified questions; informants were encouraged to elaborate on subjects they deemed important. Interviews typically took from 20 minutes to 1 hour, depending on the amount of material each participant shared. All interviews except for one took place over Zoom; one interview was conducted via phone.

All interviews were recorded except for one; detailed notes were taken for the interview that was not recorded. Otter.ai was used to transcribe recorded interviews. The interviewer then reviewed each transcript to ensure accuracy, consulting the recordings where necessary.

Interviews with regional MRH initiative participants included 13 questions, including one question each on best practices for

- Harmonization of guidelines for assessment of applications, good manufacturing practices (GMP) inspections, etc.
- Work sharing, including joint assessments and inspections
- Training and capacity building
- Information sharing
- Ensuring that timelines are met
- Sustainable funding
- Promoting ownership of initiatives at the country/REC level.

Informants were also asked about practices that regional MRH initiatives tried that did not work well, changes initiatives had made because of learning about other initiatives’ work, communication with other initiatives about their practices, major challenges, and plans for the future. The list of questions asked can be found in S1 Text.

Interviews with industry representatives included eight questions. These questions covered informants’ experience with joint assessments and inspections thus far, including best and worst practices they had identified, whether they felt industry had the chance to provide meaningful input into the regional MRH initiatives’ plans, the biggest challenges that medicines manufacturers had faced in adapting to the regional MRH initiatives’ processes, and what kind of changes industry would like to see the regional MRH initiatives make in the future. The list of questions asked can be found in S2 Text.

### Data analysis

Content analysis to categorize key issues from the data was performed by author C.A., who had no involvement in the regional MRH initiatives, including in their planning, implementation, or funding. The units of analysis were the transcripts from the interviews. First, C.A. read the transcripts to get a sense of the data collected as a whole. Second, he defined the pertinent themes for the analysis, based on the objectives of the study. Third, using the notes and transcripts, he developed categories and a coding scheme (including codes and subcodes) for the analysis, in collaboration with author K.H. Once this was done, he imported all transcripts and notes into NVIVO 12 for analysis. Each transcript or set of notes was coded, to extract information about best practices from the interviews. Inferences about best practices were drawn after analysis of the properties and dimensions of the data, with a focus on identifying relationships between themes and uncovering patterns.

### Ethical considerations

At the request of the regional MRH initiatives and the organizations supporting them, such as AUDA-NEPAD and WHO, the authors documented best practices in four African regional MRH initiatives. Everyone interviewed as part of this documentation study was either a regulator who was part of the initiatives or, in the case of industry participants, someone who worked closely with the initiatives. Given this was a programmatic study in which informants were only questioned about best practices, and all interviews were anonymized, ethical approval was not required. Individual verbal informed consent was obtained from each of the study participants.

## Results

Here we report major themes regarding best practices that emerged from the interviews of MRH initiative participants and industry representatives. The full qualitative analysis report can be found in S3 Text.

### 1. Transparency and reliability are critical

In nearly all interviews, transparency and reliability were mentioned as essential characteristics of successful regional MRH initiatives. Interviewees emphasized the importance of transparency in terms of partner countries within an initiative sharing regulatory information with each other and also in terms of the MRH initiatives sharing information about issues such as requirements and timelines with medicines manufacturers. Interviewees also stressed the importance of reliability with regard to NRAs being able to trust the quality of the work done by their partner countries, as well as with regard to medicines manufacturers being able to trust the rigor of the MRH initiatives and NRAs in making regulatory decisions and adhering to advertised processes and timelines. Here we explore different components of the initiatives that informants thought were especially important in promoting transparency and reliability.

#### Joint activities

Representatives from all regional MRH initiatives reported implementing joint assessments and inspections. Most of these participants (10 of 12) reported that the process of working together on joint assessments and inspections improved the transparency and/or rigor of regulatory processes. One respondent explained,

“It wasn’t just one country giving all the information. There was also a second country that reviewed their reports, so there was a lot of openness and ensuring that [the NRAs] learn from one another.”

–Participant, SADC

#### Timelines

In terms of best practices for improving transparency and reliability, all respondents—including MRH initiative participants and industry representatives—cited sticking to timelines, including scheduling assessment sessions ahead of time and adhering to the schedule, making timelines public, and tracking whether those timelines were being met. Eleven of 12 regional MRH initiative participants reported strides in their ability to perform joint assessments and inspections in a timely, transparent fashion. For example, respondents said,

“In terms of tracking timelines, we have come up with metric tools to measure the timelines from the submission up to the joint recommendation, and also from the joint recommendation to the market authorization at the national level.”

–Participant, EAC

“The countries have a 90-day window to allow certain country processes [after a joint assessment has been performed and a recommendation been made]. So, we are actually getting there. And in this past year, 18 months, we’ve actually been able to say that we are actively tracking and we’re able to give particular figures for the indicator.”

–Participant, SADC

ZAZIBONA, SADC’s regional MRH initiative, was cited by multiple participants as a model for scheduling assessments and communicating clear timelines. ZAZIBONA creates an annual schedule for joint assessments well in advance and adheres to it, so that it is clear to manufacturers when their applications will be reviewed.

Overall, however, industry representatives reported considerable room for improvement when it comes to the transparency of initiative timelines and processes.

“The timelines are not so reliable. What is written down is very attractive, but often we find that some of the pathways are taking much longer.”

–Participant, Industry

“There are some specific country requirements that follow this joint assessment decision. So actually, not just prolonging the timelines but adding to the complexity, because you’ve submitted a dossier, and then you need to submit additional documents or additional samples or additional whatever the further requirements are.”

–Participant, Industry

One regional MRH initiative participant explained,

“It’s the countries that are delaying. There is the regional economic bloc and there is the NRA. Those two are different. So, the marketing authorization or registration is offered by the national agencies, not by the regional economic blocs, so there’s always a delay after a regional decision.”

–Participant, IGAD

In addition, some regional MRH initiatives found themselves having to reschedule and postpone joint assessment sessions in light of the participants’ schedules, which made offering transparency and reliability to applicants more challenging.

#### Information management systems

All respondents from regional MRH initiatives reported that well-functioning information management systems are key for providing regulatory transparency, especially when multiple NRAs must work together in an initiative. Figuring out information management systems can be a quagmire, however. Indeed, all respondents reported concerns with the functionality of their current system. Specifically, participants from the EAC explained that individual NRAs developed their own information management systems. This meant that the systems varied in functionality and quality and were also not compatible with one another.

“Because everyone has got their own system, then you need this common platform whereby you can share information. So, this has been a daily challenge.”

–Participant, EAC

Participants from other regional MRH initiatives reported similar problems with their systems for sharing information.

“The communication lag is too long. To me, I think that’s the biggest challenge.”

–Participant, ECOWAS

“IGAD has an information sharing portal… But the problem that we have is management of that database because most countries take time to upload… it’s not automatic. So, it’s not linked to what is being done at the NRA.”

–Participant, IGAD

### 2. Reliance is essential for smart regulation

Given limited time and resources, the majority of informants emphasized reliance as a best practice. Here, the term reliance refers to regulators leveraging the output of other regulators whenever possible, so that they can concentrate on carrying out regulatory activities that cannot be undertaken by other authorities, such as in-country pharmacovigilance and market surveillance or oversight of local manufacturing and distribution [16]. Examples of reliance include willingness to adopt existing guidelines and using regulatory assessments performed by other NRAs or regulatory authorities. With regard to adopting existing guidelines and documents, 11 out of 12 respondents from regional MRH initiatives reported that relying on work done by other regional MRH initiatives and global institutions such as WHO was a helpful practice, and one that they had employed in their initiatives.

“When we started, we didn’t really have harmonized guidelines as such, but we wanted to start… So, we just jumped in and started doing the work and used the WHO guidelines. Within a year, we developed and adopted CTD [common technical document] guidelines in the SADC region.”

–Participant, SADC

“One thing that we quickly picked up was the issue of wanting to get our guidelines sorted. We approached EAC and asked if they could actually assist with whatever they have. And then we looked at whatever was in their compendium and then tried to build up from that.”

–Participant, SADC

“One of the things which we first did was to harmonize the documents, the guidelines, and the forms—the working tools that we use. And because of that, we were able to get best practices, the best practices in the world, because we were benchmarking on documents that have already been used by other more developed regulatory agencies.”

–Participant, IGAD

Nine out of 12 participants, including respondents from every regional MRH initiative, reported that relying on regulatory assessments and inspections by other NRAs was also a helpful practice, and eight participants described implementing such reliance in their initiatives. Many respondents reported an increasing level of trust within the initiatives over time, which facilitated NRAs relying on regulatory assessments performed by other NRAs in their region.

“You now hear a lot of countries happier to say, ‘Well, if [country X] has done it, then I don’t have to look at it so carefully. Because I know the standards with which [country X] is doing it’… Even if an application did not come through the central system, even without a written memoranda of understanding to say that we are going to rely on the data, we are a lot more comfortable saying that I can rely on what this country has done.”

–Participant, ECOWAS

“Small, small countries… It might not make sense for them to actually have a full-fledged NRA. So they might then choose certain functions and then rely on everyone else within the region.”

––Participant, SADC

Another helpful practice with regards to reliance, mentioned by eight of 12 respondents, including from all regional MRH initiatives, was benefiting from partnership with other NRAs in their initiative by capitalizing on their relative strengths. Seven of 12 respondents, again including participants from all initiatives, reported that they had already benefited from the strengths of partners. Sample comments included:

“We were borrowing the strength from other countries. Like Uganda was a bit more advanced in GMP inspections, because they started much earlier. And well, we would adopt reports from that country, the ones they did.”

–Participant, EAC

“You may find that not all countries have the same expertise. Kenya has some strength, especially in pharmacovigilance and even registration of medicine… And with harmonization, we share this expertise. Because once an evaluation is done via what we call a common procedure, then we would adopt it in other countries, so they don’t need their own.”

–Participant, EAC

One particular strategy that five of 12 respondents mentioned they had implemented in their regional MRH initiative and found helpful, including participants from three out of four of the initiatives, was dividing into working groups devoted to specific issues, each led by a different NRA according to their strengths.

“We broke into the different working groups. We had the registration working group, the clinical trials working group, the information management systems working group, and others…. What we did was we looked at our strengths… And then as well, we also had some people who actually also were made to join working groups, because maybe their capacity was slightly lower.”

–Participant, ECOWAS

### 3. Multiple successful strategies for NRA capacity building have been identified

Many informants said that one of the major challenges for the regional MRH initiatives was the different maturity levels of the various member countries. To rely on one another’s work, each NRA must have a base level of maturity that allows its partners to trust its decisions. In other words, many of the initiatives found that capacity building was necessary to build a foundation for reliance. A respondent described this challenge:

“When you’re doing assessments jointly, the levels of advancement are different. Sometimes there are a lot of back-and-forth exchanges amongst ourselves, and sometimes that could prolong the process.”

–Participant, EAC

For example, in the EAC, Rwanda established an autonomous NRA in 2018, and it has since used its membership in the EAC’s regional MRH initiative to improve its regulatory maturity. Here, we describe several strategies that informants believed were helpful for building capacity and hence facilitating reliance.

#### Twinning

Five participants, including respondents from all four regional MRH initiatives, mentioned “twinning” as a best practice for capacity building within initiatives. Four of these participants, again from all four regional MRH initiatives, described implementing this strategy and reported it helped improve the regulatory capacity of less mature NRAs, building trust within the initiatives.

“In 2015, we began what we call mentoring and twinning, where the countries visit, spend a month. They moved across the region to spend some time, 2 weeks, 3 weeks, or a month with each other, the French moving to the English, the English moving to the French. They just look at what each other is doing, learn from them, build that trust and confidence. So, it was a time for them to understand each other and to work as a region and integrate themselves.”

–Participant, ECOWAS

#### Capacity building during joint assessment sessions

Six of 12 informants, including representatives from all MRH initiatives, observed that the simple act of participating together in joint assessment and inspection sessions can be a very effective training method and reported having benefited from this type of capacity building. One observed,

“I suppose that’s the only way of getting people together, to work together, the people with stronger capacity taking along the countries which have weaker capacity… We made sure we incorporated countries that didn’t have experts but should be part of the process, even as observers. So, I think that was one of the best practices, for the stronger countries to bring up the weaker countries as a form of capacity building.”

–Participant, ECOWAS

Nine of 12 participants, including representatives from all four regional MRH initiatives, reported that providing formal training opportunities for initiative personnel was a helpful practice that they had implemented. Sometimes these training sessions took place during the joint assessment sessions themselves:

“Another good practice that we adopted was that on the first and second day [of a joint assessment session], for 2 hours, we get someone to present on a topic, even if it’s someone external… So, each session incorporates a particular training. And those slides are then uploaded into MedNet, and then they are accessible to the different countries.”

–Participant, SADC

“During the joint session, we used to have 1 or 2 days of capacity building by a senior expert from the Swiss Agency for Therapeutic Products to train assessors on how to evaluate clinical data in the dossiers.”

–Participant, EAC

#### WHO global benchmarking assessments

Four of 12 participants, coming from two of the four regional MRH initiatives, mentioned that having member countries participate in WHO’s global benchmarking assessment process was helpful for building trust and improving regulatory capacity within the initiatives. The same participants reported having implemented benchmarking within their initiatives. Informants acknowledged that organizing member countries to complete these comprehensive assessments (e.g., in the form of regional benchmarking workshops) was not always easy, but it was worthwhile. One respondent explained,

“For SADC, we have so many countries, so you can imagine being at different levels… For those that had a certain [WHO] maturity level, we wanted them to progress from 2 to 3, and those that were at the lower level to progress to another level. I think, what helped was also the benchmarking, self-benchmarking, so that you know at what level you are, and then you also develop the institutional development plans. We are now following up on the implementation of those institutional development plans that were done at country level.”

–Participant, SADC

Tanzania’s NRA (a member of the EAC MRH initiative) reached Maturity Level 3 in 2018, and Ghana’s NRA (a member of the ECOWAS MRH initiative) reached Maturity Level 3 in 2020, shortly before this study was conducted. These were the first NRAs in Africa to achieve this level of regulatory maturity.

### 4. Communication between heads of agencies is essential

One helpful practice that four of 12 informants, from two of four of the initiatives, mentioned implementing was frequent communication between the heads of NRAs in an REC, as well as frequent communication between other key staff involved in joint activities. This communication took place both in the form of institutionalized heads of agencies sessions or informal exchanges. Respondents reported that the regional MRH initiatives have brought heads of agencies together in a way that had never happened before, with wide-ranging benefits.

“Since there were four of us, we rotated around each of the four countries to [run Heads of Agencies sessions]. And when we came to your country, you chaired the Heads of Agencies session, and you ran the meeting, so that you also took ownership… And that has created a real close-knit family of heads of agencies. Even when we have other issues that are outside assessments and things, we are always in touch.”

–Participant, SADC

“In our case, we have a WhatsApp group, everyone on one platform, heads of agencies, just to communicate on a regular basis on what needs to be done in our region, in our harmonization program.”

–Participant, EAC

“Now we could call each other anytime. It’s quite different from the way it used to be before.”

–Participant, EAC

### 5. Cooperation at the regional level is not possible without leadership at the NRA level

Nine of 12 informants, from all the regional MRH initiatives, noted that enthusiastic leadership at the country level was helpful in ensuring that the initiatives have the support they need to succeed. Six of the 12 reported observing such leadership in their own initiatives. One explained,

“Political will is critical… And it has to start from the top. As the head of agency, you need to show that you are interested, you need to show that with examples, that you are really keen on what everyone is doing.”

–Participant, EAC

Another respondent noted that one threat to such enthusiastic leadership is the belief of some NRA leaders and staff that the regional MRH initiatives compete with the NRAs for resources. If the leadership of a country believes that an initiative threatens its funding, it is not likely to devote the staff and resources needed for the initiative to thrive.

With regard to leadership, a helpful practice highlighted by three of 12 informants, from 2 of the regional MRH initiatives, was to ensure that no initiative depended disproportionately upon just one person or one country, as the initiative wouldn’t be sustainable over the long term. All three informants reported that their initiatives were taking steps to distribute power across multiple centers.

“[MRH initiatives] should not try and create centers of power in one country. They should not, for example, make one agency become the super-regulator in the home, because it brings political issues; they should try and distribute the workload among different NRAs, among different countries, so that at least there is capacity.”

–Participant, IGAD

### 6. Sustainable funding remains challenging

Many of the regional MRH initiatives started with support from donors, but 11 of 12 respondents, including participants from every initiative, believed that in order to be sustainable, permanent funding mechanisms must be established. All of these informants touched on the challenges of developing such a sustainable funding mechanism. Only five of the 12 respondents, from two of the initiatives, reported implementing coordination or top-up fees, to help support their initiatives’ activities. However, participants from other initiatives reported progress toward, or at least plans for, charging joint assessment or GMP inspection fees to cover the initiatives’ work.

“In EAC, the heads of agencies approved a sustainability plan. We are proposing a top-up fee to be charged over and above the national fees, so that if somebody wants to use this regional system, they pay a top-up fee, and then that top-up fee will be used to run the regional system.”

–Participant, EAC

“In future, we’re looking at having a coordination fee. So that if [applicants] say they want a joint assessment, and they want us to convene a meeting, we can convene say five meetings in a year.”

–Participant, IGAD

The system for funding joint activities that has been established by SADC may represent a best practice. When SADC’s regional MRH initiative, ZAZIBONA, began operating, it did so without donor funding. As a result, it had to identify a means for funding joint assessments and inspections right away.

“We have been conducting our joint inspections on a cost-recovery basis. Right from the get-go, our joint inspections have not been supported by partner funding.”

–Participant, SADC

“Right from the beginning, what we had talked about was… if a product comes through the ZAZIBONA mechanism, of apportioning some of the application fees towards the initiative. Because we’re looking ahead, we’re saying donors are not going to be around forever.”

–Participant, SADC

In addition, some informants mentioned that a shift to virtual joint assessment sessions, driven by the pandemic, might offer a more cost-effective way of holding these meetings in the future.

Industry informants stated that they were receptive to the regional MRH initiatives charging top-off or coordination fees, so long as the fees were accompanied by sufficient benefits for applicants. However, these same informants believed that, currently, these types of benefits were too often lacking.

“One thing which is a big ask from the industry would be predictable timelines, and also reliable processes and timelines… More like a guaranteed level of service that applicants could get. If you use that as a way to make the argument, it could mean that this system could lead to a little bit of increase of the fee… This kind of guaranteed outcome is what there needs to be, watertight. That you pay this kind of fee, or it could even be, let’s say graduated, it could potentially also have something like a Fast-Track kind of thing, depending on the kind of product which is being applied for. But in the end, these deliverables need to be there.”

–Participant, Industry

### 7. Industry has important insights

Most industry informants believed that the regional MRH initiatives hold promise. However, although some reported already observing benefits to applicants, others stated that, in terms of time and resources saved by using the joint application and inspection processes, the initiatives were still very much a work in progress. Respondents observed,

“Some of the manufacturers that participated in the joint assessment procedures and also joint inspections, we noticed that it is, in general, beneficial to join those pathways, because we see overall time saving and then, in some cases, also procedures that are more transparent and more predictable compared to the national ones. And what we also see as a benefit is that the timelines are shortened through the regional joint assessment procedures compared to national.”

–Participant, Industry

“Overall, [the regional MRH initiatives have] been a positive development in terms of having the process of assessment and inspections. One of the positive things, of course, would be guideline development, that guidelines which never used to exist now are there in one way or another. It’s a question of whether they have been fully implemented and to what extent they are being implemented. That is something else.”

–Participant, Industry

“The unpredictable timelines, in terms of planning of the joint assessment, at different levels, timelines for dossier submissions, timelines for dossier review, timelines for queries, and timelines to get back to manufacturers on the queries or timelines for the end, receiving the approval and the authorization at national level, can be done a bit better.”

–Participant, Industry

Industry informants made it clear that they valued opportunities to work with the regional MRH initiatives to set their agendas and develop processes, and they expressed a desire to take a greater role in providing input in the future.

“IFPMA [International Federation of Pharmaceutical Manufacturers & Associations], since the start of the AMRH initiative, was part of the discussions at the steering committee level and is a partner of the initiative. Through this, there is continuous engagement with the key players of the different regional Secretariats. So, we would take any opportunities, face-to-face meetings, when we would have the steering committee meetings, but also sometimes the scientific conference organized by NEPAD to ensure that we would meet all these regional Secretariat representatives, discuss our challenges, discuss our successes, and try to also see where we can together improve, and harmonization of our work in the regulatory fields.”

–Participant, Industry

“Sometimes the initiative already has their agenda, and inviting industry to participate is really just rubberstamping. Their feedback isn’t really wanted.”

–Participant, Industry

“We see the challenge that we are not so well informed of what is in the pipelines for these different regional Secretariats. We would really recommend that they could share in advance a work plan for the coming years… Sometimes when we share our comments it is through our trade associations, but we don’t know at the end, for example, the process… We don’t know how the feedback is used, or how industry could better support this regional initiative going forward.”

–Participant, Industry

“The issue with the regional guidelines, when we were informed of these guidelines, sometimes late, we would have maybe 2 weeks to react… The [region X] variation guidelines, we knew that they would be coming, we knew that they would be having a consultant working on them. But the contract with the consultant was so short that at the end, no time was left for stakeholders to provide comments. And even if we could provide comments, then the comments were not maybe considered, because they really wanted to wrap up the project, which is not a good practice.”

–Participant, Industry

Industry informants mentioned several specific suggestions for regional MRH initiatives, moving forward:

“The scope of the joint assessments, the list of products, is too restrictive… There should be a mechanism to include products of interest and also allow innovative medicines, rather than just old products, to be in the pipeline.”

–Participant, Industry

“Definitely the WHO guidelines and public consultation process is something that we would recommend. And also, we’d like to encourage the RECs to develop a work plan for a couple of years or 5 years, not only for new guidelines, but also for any guidance that would be getting old and need to be revised. And also maybe a clear indication and more transparency on the regional websites of the Secretariat when they exist, of what is the status of the guideline in terms of implementation, whether it’s adopted, whether it’s implemented in all countries in the region—this would be really helpful.”

–Participant, Industry

Importantly, only two of the 12 participants from regional MRH initiatives, including representatives from two of the four initiatives, mentioned seeking industry participation in the creation of guidelines and policies as being a helpful practice, and only one participant reported taking meaningful steps to promote such participation in their initiative. Thus, finding an appropriate role for industry to play with regards to the work of the MRH initiatives may represent a promising opportunity to explore in the future.

## Discussion

In this study, we interviewed key participants from four of Africa’s regional MRH initiatives—EAC, SADC, ECOWAS, and IGAD—and also representatives from the pharmaceutical industry, seeking to identify best practices for optimizing the regulatory environment on the continent. In doing so, we also identified continued challenges faced by the MRH initiatives, which launched between 2012 and 2017. Here, we discuss the findings that we believe may be most helpful to those involved in the AMRH initiative, as well as to key stakeholders in other MRH initiatives around the world.

Informants made it clear that one of the best practices is for regional MRH initiatives to maintain balance between capitalizing on the expertise of more mature NRAs and strengthening the capacity of less mature members. On the one hand, regional MRH initiatives are based on the principle of reliance, allowing less mature NRAs in an REC to benefit from the expertise of more mature NRAs. On the other hand, regional MRHs serve to strengthen every participating NRA. It is clear from the interviews that training sessions, twinning and the exchange of workers between NRAs, and the process of work sharing itself provide opportunities for less mature NRAs to build capacity. In particular, the transfer of people between sites was reported to be important, as it fosters the transfer of implicit or tacit knowledge, as well as explicit knowledge, needed to implement best practices [13]. In addition, many participating NRAs reported using the WHO’s Global Benchmarking Tool [11, 12] to assess their regulatory maturity and identify areas in need of improvement. For example, Tanzania’s NRA reached Maturity Level 3 in 2018, and Ghana’s in 2020. After our interviews concluded, additional African NRAs also achieved Maturity Level 3, including those of Nigeria (a member of the ECOWAS regional MRH initiative), South Africa (a member of ZAZIBONA, the SADC regional MRH initiative), and Egypt. A robust process of self-benchmarking at the regional level, in which NRAs participate together and can see the practices of their peers, helps drive everyone toward a higher level of performance and maturity. In addition, having a system in place to track each NRA’s progress toward implementing its institutional development plan, created in response to its self-benchmarking results, keeps each country abreast of the improvements being made by their neighbors.

Improving the capacity of all members of an MRH initiative is key for building trust within the region, so that true collaboration can occur. For an initiative to succeed, each member must be comfortable relying on the work performed by the others. Within such a system, different NRAs can play a leadership role for areas in which they excel. When consensus is present that a given NRA is especially qualified in a particular area, all members of a regional MRH initiative can feel comfortable letting that NRA take the lead in a joint assessment. This is more efficient than requiring multiple leads, which some MRH initiatives have done. Conversely, as respondents pointed out, spreading the responsibility for joint activities across multiple NRAs and personnel is key for the stability of MRH initiatives over the long term.

The regional MRH initiatives are also flexible enough to allow individual countries to select the regulatory structures that make the most sense for them. For example, Rwanda, a member of the EAC’s MRH initiative, established an autonomous NRA in 2018 and has since pushed hard to achieve excellence. By contrast, as a participant from ZAZIBONA mentioned, some other countries have decided not to establish their own autonomous agencies because they are so small. In addition, being part of a network allows the NRAs within a regional MRH initiative to share the workload in a manner that reflects their strengths. For instance, as interviewees mentioned, in the EAC, each country has a different strength: Tanzania focuses on product evaluation and registration; Uganda on GMP inspections; Rwanda on information management systems; Burundi on clinical trials; South Sudan on policy, legal, and regulatory reforms; Zanzibar on quality management systems; and Kenya on pharmacovigilance [3, 17].

As described above, strong leadership stemming from the heads of NRAs emerged as a key best practice. Because donor funding cannot be relied on indefinitely, having strong commitment at the NRA level is the only way to make regional MRH initiatives sustainable. Interviewees from ZAZIBONA, for example, mentioned that the initiative began without donor funding, relying on enthusiastic heads of NRAs who were willing to organize activities. ZAZIBONA has also devised strategies for continuing basic joint activities when donor funding ceases. Of note, multiple informants mentioned that the pandemic showed that virtual assessment sessions worked well and cost much less than in-person sessions, since they obviated the need for travel. Going forward, holding more virtual sessions might be an important way to keep costs down and alleviate scheduling problems. Conducting virtual GMP inspections is more challenging, but individual NRAs and regional MRH initiatives are developing ways to make even this feasible. At the same time, face-to-face meetings may play a critical role in building trust and strengthening relationships between regulators, as well as between regulators and manufacturers. Therefore, it seems likely that regional MRH initiatives might hold a mix of face-to-face and virtual sessions and inspections in the future.

A major challenge that emerged from the interviews was coordination across RECs. Take, for example, information management systems. An efficient means of sharing information between NRAs is essential to the success of the MRH initiatives. However, interviews revealed that none of the regional MRH initiatives has yet developed a functional information management system to facilitate such sharing. In the EAC, individual countries each developed their own information management systems in parallel, rather than committing to developing a single functional regional system. This resulted in good systems in some countries, poor systems in others, and the absence of a regional system that could be used by all members, with no clear plan for moving forward in terms of connecting the systems used by the various NRAs.

The industry participants involved were generally supportive of the regional MRH initiatives, though they offered substantive constructive feedback aimed at future improvements. Major suggestions included increasing the transparency and predictability of timelines, providing opportunities for industry to consult on important issues, and expanding the scope of products eligible for joint assessment. Multiple participants noted that ZAZIBONA’s approach to organizing assessments has been especially successful. ZAZIBONA creates an annual calendar for assessments and adheres to the timelines, so that it is clear to manufacturers when their applications will be reviewed. In addition, the less politicized the joint assessment process is, the more efficient it becomes. Limiting attendance to the parties integral to performing assessments makes it easier to schedule regular sessions and to refrain from having to reschedule due to conflicts.

We note that the challenges faced by the AMRH initiative are not unique; for context, consider that years ago, the EMA confronted many of the same problems. Although the EMA officially launched in 1995, efforts to start harmonizing medicines regulation in Europe started much earlier, in the 1960s [18]. In its first year, the EMA received only eight marketing authorization applications through its centralized pathway from manufacturers who were not obliged to use it; it took years for the numbers of optional applications to grow to their current numbers, for both this pathway and the system’s mutual recognition pathway [18].

Early on, marketing authorization applicants had many complaints about the European Union (EU)’s mutual recognition pathway for marketing authorization, which is most similar to the set-up of the AMRH initiative’s joint assessment procedure [18]. The young EMA also struggled with uneven contributions from member states, in terms of which regulatory agencies served most often as the rapporteurs who handled assessments [19]. Finally, in its early years, the EMA was criticized for a lack of rigor in its assessments, including variable presentation styles, lack of clarity, and failure to conform to stated aims [20].

A commitment to transparency helped the EMA overcome its early growing pains. One key step it took that many national bodies in Europe had not taken prior was to produce a public assessment report for each licensing decision [20]. By making its work public, the EMA was able to be accountable, collect constructive criticism, and improve.

Another instructive lesson for regional MRH initiatives may be that, by the 1980s, it had become clear in the EU that harmonizing national rules was not enough. Instead, a system of binding decision-making had to be introduced to achieve the creation of a unified European market [21]. Someday, this may become a reality across Africa as well.

This study has several important strengths. First, it included in-depth interviews with participants from four different regional MRH initiatives in Africa, allowing identification of best practices that emerged in the different institutions. The individuals interviewed were the people running the regional MRH initiatives. As such, they have greater familiarity with the initiatives’ best practices and challenges than anyone else. In addition, the authors who interviewed participants and analyzed the data had no involvement in the regional MRH initiatives, including in their planning, implementation, or funding, promoting an objective analysis of best practices. The fact that participants shared both positive and negative feedback about the initiatives demonstrates that they felt comfortable being honest about what worked well and what did not work well.

This study also has several important weaknesses. First, our semistructured interview format was implemented to ensure that all respondents were presented with identical prompts and that all topics that we believed were important would be discussed. However, in some cases, a respondent might have believed that a practice was helpful, or their initiative may have even implemented such a practice, but the practice was not mentioned in the interview. For example, given the nature of these initiatives, presumably all participants believed that relying on regulatory activities performed by other NRAs is helpful. Even so, only nine of 12 respondents mentioned this being a helpful practice in their interviews. They may have simply taken for granted that this practice was helpful and thus not explicitly mentioned it. Because three representatives were interviewed from every initiative, we believe that even with some omissions of this type, the data we collected should capture most best practices.

Second, the regional MRH initiatives we investigated were not comparable in all ways. For example, some initiatives were established years earlier than others, the amount and type of their funding differed, and each was unique in terms of language, politics, and legal and regulatory frameworks. This might limit the generalizability of any best practice identified in a single initiative. However, our theoretical framework, which took into account how widely practices were viewed as effective and also adopted, implemented, and maintained, helped ensure that our focus remained on fairly generalizable best practices.

Finally, in this explorative, qualitative project, we did not seek to confirm the effectiveness of best practices by determining whether they were associated with superior outcomes. Future studies may seek to investigate whether a given practice, such as scheduling a year’s worth of assessments in advance and adhering to that schedule, is associated with desired outcomes, such as meeting advertised timelines and securing a higher volume of applications, across regional MRH initiatives. Such work will grow easier as initiatives mature and produce the requisite quantitative data, particularly if the same key performance indicators are collected for comparison. Verifying whether the best practices identified in this study are actually associated with desired outcomes would strengthen the quality of available evidence [22].

In conclusion, since the first African regional MRH initiative began in 2012, a wealth of knowledge regarding best practices and approaches to dealing with common challenges has accumulated on the continent. As regional MRH initiatives mature, it should be possible to determine with greater confidence which of the best practices identified here are most helpful. Historically, many practices that have been found to be effective in an original, highly motivated, or more advantaged setting have been found to be less effective when implemented in a more typical setting [15]. Therefore, determining which practices are consistently effective across initiatives and across time will be an important area for future study. We hope that the information we have shared in this article can benefit regional MRH initiatives inside and outside of Africa, ultimately helping them to accelerate access to quality, safe, effective medical products.

## Supporting information

S1 Text Questions asked during interviews of MRH initiative participants. (DOCX)

S2 Text Questions asked during interviews of industry representatives. (DOCX)

S3 Text AMRH initiative best practices report. (PDF)

## Abbreviations

AMRH: African Medicines Regulatory Harmonization
AUDA-NEPAD: African Union Development Agency–New Partnership for Africa’s Development
BMGF: Bill & Melinda Gates Foundation
CTD: Common technical document
EAC: East African Community
ECCAS: Economic Community of Central African States
ECOWAS: Economic Community of West African States
EMA: European Medicines Agency
EU: European Union
FCDO: Foreign, Commonwealth and Development Office
GMP: Good manufacturing practices
IGAD: Intergovernmental Authority on Development
MRH: Medicines regulatory harmonization
NRA: National regulatory authorities
REC: Regional economic communities
SADC: Southern African Development Community
WHO: World Health Organization
