# Supplementary material for: Best practices in the African Medicines Regulatory Harmonization initiative: Perspectives of regulators and medicines manufacturers
Source: PLOS Glob Public Health. 2023 Apr 26;3(4):e0001651. doi: 10.1371/journal.pgph.0001651 (PMC10132525; doi:10.1371/journal.pgph.0001651)
Supplement: S1 Text — (DOCX) [file pgph.0001651.s001.docx]

**AMRH Best Practices Interview Questions**

1. Since the initiative began in your region, what are some of the practices that have been most helpful for medicines regulatory harmonization (ie, harmonization of guidelines for assessments, inspections, applications, etc.)
2. What has worked well as the countries have worked together to assess products or done inspections jointly? What about for work sharing?
3. What about for training or capacity building? What have been the best practices?
4. What about for information sharing? What have been the best practices?
5. What about for ensuring that recommended products are authorized across countries according to the agreed upon timelines? What have been the best practices?
6. What has your progress toward sustainable funding been like so far?
7. From an ownership perspective, what are some examples from your region of countries demonstrating a level of ownership of the initiative?
8. Are there any things the initiative has tried that did NOT work out well? Things you would encourage other regions to avoid?
9. What changes has your initiative made as a result of learning from the experiences of other medicines regulatory authorization initiatives?
10. Are there any things that other initiatives are doing that you would like to try in your initiative, but have not been able to so far? Why?
11. Do you feel like you have a pretty good idea about the kinds of things other medicines regulatory harmonization initiatives are doing?
12. What have been the biggest challenges your initiative has faced?
13. What are you excited about when you think about the initiative’s future? Are there any important changes that you are working on now?
