# Supplementary material for: Best practices in the African Medicines Regulatory Harmonization initiative: Perspectives of regulators and medicines manufacturers
Source: PLOS Glob Public Health. 2023 Apr 26;3(4):e0001651. doi: 10.1371/journal.pgph.0001651 (PMC10132525; doi:10.1371/journal.pgph.0001651)
Supplement: S2 Text — (DOCX) [file pgph.0001651.s002.docx]

**Interview questions for industry participants about regional medicines regulatory harmonization initiatives in Africa**

1. Has the process of assessment and inspection gotten easier for industry as a result of the harmonization of technical requirements/guidelines for marketing authorization? Have you noticed any best practices for this type of harmonization on the part of the regional initiatives (EAC, SADC/ZAZIBONA, ECOWAS, etc)?
2. What has your experience of joint assessments and inspections been like so far? Have you noticed any best practices—things that worked especially well from the industry perspective? Anything that has not worked so well?
3. After joint assessments, has it been your experience that recommended products are authorized by countries according to the agreed upon timelines? Are any regional initiatives doing this particularly well?
4. Do you think most drug manufacturers are willing to pay higher fees for joint assessments and inspections than they would pay if they applied to each country individually? What would they expect for higher fees in terms of greater coordination and convenience?
5. Are there any practices of regional initiatives that you have found do not work well for industry? Are there things you would encourage the initiatives to avoid?
6. Do you feel like any of the regional initiatives have done a good job at seeking industry feedback when they are creating plans or developing guidelines that will affect industry?
7. What have been the biggest challenges for drug manufacturers in adapting to changes by the new regional initiatives, whether it involves newly harmonized guidelines, joint assessments or inspections, etc?
8. In an ideal world, what kind of changes would you like to see the regional initiatives make? What would improve access to medical products by streamlining the regulatory process?
