# Supplementary material for: Best practices in the African Medicines Regulatory Harmonization initiative: Perspectives of regulators and medicines manufacturers
Source: PLOS Glob Public Health. 2023 Apr 26;3(4):e0001651. doi: 10.1371/journal.pgph.0001651 (PMC10132525; doi:10.1371/journal.pgph.0001651)

The AMRH Initiative Best Practices Report

Submitted by:
Collins Agaba I Consultant

Table of Contents

Abbreviations ... iii
Introduction ... iv
Methods ... v
OBJECTIVE 1: TO DOCUMENT LESSONS LEARNED BY REGIONAL MRH INITIATIVES, ESPECIALLY REGARDING BEST PRACTICES ... 1
Medicines regulatory harmonization ... 1
Work and information sharing ... 3
Training and capacity building ... 7
Sustainable financing ... 9
Country ownership of regional harmonization programs ... 10
Ensuring that recommended products are assessed and authorized within the agreed upon timelines ... 12
Consistency of guidelines at the country level ... 15
OBJECTIVE 2: TO EXPLORE THEMES SUCH AS PRACTICES THAT DID NOT WORK WELL, THE INDUSTRY EXPERIENCE, BEST PRACTICES, CHALLENGES, AND FUTURE DIRECTIONS ... 15
Practices that did not work well ... 15
Openness of regional initiatives to change, and learning that occurred between initiatives ... 16
Whether the process of assessment and inspection has gotten easier for industry as a result of regional MRH initiatives ... 17
How industry has experienced regional MRH initiatives' joint assessments and inspections ... 18
Willingness of industry to pay higher fees for joint assessments and inspections than for individual country assessments and inspections ... 19
Whether industry feels its feedback has been included when regional MRH initiatives create plans or develop guidelines ... 20
The biggest challenges industry has faced in adapting to changes made by the regional MRH initiatives ... 21
Changes that industry would like to see the regional initiatives make ... 22
Key Best Practices for MRH Initiatives ... 23
"Twinning" between more and less mature regulatory authorities ... 23
Joint assessments and inspections ... 23
Expert working groups ... 25
Benchmarking as a way to build trust and improve regulatory capacity ... 26

Challenges Faced by the Regional MRH Initiatives 26
Lack of resources and time 26
Staff turnover 27
Language barriers 27
Information sharing 28
Lack of transparency in the assessment calendar 28
Future Prospects for Regional MRH Initiatives 29
Developing functional information sharing systems 29
Capacity building 29
Funding 30
Expansion to new areas 30
Word frequency cloud 31

Abbreviations

AMRH	African medicines regulatory harmonization
API	Active pharmaceutical ingredient
AUDA-NEPAD	African Union Development Agency–New Partnership for Africa's Development
CTD	Common technical document
EAC	East African Community
ECOWAS	Economic Community of West African States
GMP	Good manufacturing practice
ICH	International Council for Harmonisation of Technical Requirements for Pharmaceuticals for Human Use
IFPMA	International Federation of Pharmaceutical Manufacturers & Associations
IGAD	Intergovernmental Authority on Development
MRH	Medicines regulatory harmonization
NMRA	National medicines regulatory authority
OCEAC	Organization of Coordination for the Fight against Endemic Diseases in Central Africa
REC	Regional economic community
SADC	South African Development Community
WAHO	West African Health Organization
WHO	World Health Organization

Introduction

This assessment of the African Medicines Regulatory Harmonization (AMRH) initiative utilized a qualitative method of enquiry to deliver on two key objectives:

(1) To document lessons learned by regional MRH initiatives, especially regarding best practices in the areas of MRH, work-sharing, training and capacity building, disseminating information within and between national medicines regulatory authorities (NMRAs), sustainable financing, and country ownership of regional MRH initiatives. In addition, this assessment explored whether recommended products were authorized across countries within the agreed upon timelines, whether regional MRH activities were redundant with activities by other institutions, and how efficient regional and national harmonization processes were.

(2) To explore themes such as practices that did not work well, how open the regional initiatives were to change, learning that occurred between the initiatives, whether the process of assessment and inspection has gotten easier for industry as a result of regional MRH initiatives, how industry has experienced the regional initiatives' joint assessments and inspections, and whether industry is willing to pay higher fees for joint assessments and inspections than for individual country assessments and inspections. Other aspects of the industry experience that were assessed in this evaluation include whether industry feels its feedback has been included when regional MRH initiatives create plans or develop guidelines, the biggest challenges industry has faced in adapting to changes made by the regional MRH initiatives, and changes that industry would like to see the regional MRH initiatives make.

Key best practices documented in this report include reliance, working together, utilization of existing regulatory guidelines and standards, openness to change, and willingness to learning from other initiatives and institutions. However, suboptimal legislative frameworks and administrative procedures, challenges in information sharing, and difficulties with timing tended to cripple the work of regional MRH initiatives. Other critical issues included lack of clarity in the submission requirements for products being reviewed by regional MRH initiatives and conflicting interests across the different countries and regional economic communities (RECs). In some countries, conflicting interests occurred due to NMRAs' fear of losing funding or suppliers imposing adoption of their own systems. Our results show that work sharing has helped MRH initiatives begin to ease regulatory processes, despite challenges such as lack of a uniform information sharing platform. Capacity building and training efforts led by experts (local and international) were reported to work well, especially when they incorporated both theory and practice. However, sustainable funding and staffing still pose a challenge for the regional MRH initiatives. Despite these difficulties, different regional mechanisms facilitate REC ownership of the

programs, including rotating leadership roles, holding meetings and open discussions, and engaging in cost-sharing at the country level. The future of the AMRH initiative appears bright, as evidence in the subsequent sections indicate.

Methods

To conduct this evaluation, interviews were conducted with key informants from four regional MRH initiatives: the East African Community (EAC), South African Development Community (SADC), Economic Community of West African States (ECOWAS), and Intergovernmental Authority on Development (IGAD). For each regional MRH initiative, 2 heads of NMRAs and one member of the Secretariat were interviewed. In addition, four industry informants were interviewed, 3 participants in a group interview setting that International Federation of Pharmaceutical Manufacturers & Associations (IFPMA) representatives helped organize and one participant in an individual interview. All interviews followed a semi-structured format, consisting of open-ended, pre-specified questions; informants were encouraged to elaborate on subjects they deemed to be important.

All interviews were recorded. Interviews were then coded and analyzed using NVIVO to extract themes. The results of this qualitative analysis are presented here.

OBJECTIVE 1: TO DOCUMENT LESSONS LEARNED BY REGIONAL MRH INITIATIVES, ESPECIALLY REGARDING BEST PRACTICES

Medicines regulatory harmonization

Discussion with various study participants highlighted a number of lessons learned in relation to MRH. The majority of informants emphasized that since they started participating in regional MRH initiatives, reliance, working together, willingness to adopt already existing international guidelines (mainly from the World Health Organization [WHO] and the International Council for Harmonisation of Technical Requirements for Pharmaceuticals for Human Use [ICH]), openness to change, and willingness to learn from one another have been instrumental in making progress toward streamlining regulatory processes. For instance, reliance has enabled regulatory organizations to use regulatory assessments from other authorities, without having to repeat the whole process. Lessons learned and best practices relevant to MRH are stressed in the quotes below:

> We have definitely worked together, we have produced some sort of harmonization, but it is still not like a complete harmonization. We have produced some draft guidelines that countries can adopt. When it comes to registration, we have had one […] who has actually tried to organize, and they have actually done that successfully for registration. (Participant, ECOWAS)

> This is not what will probably be acceptable to most people. When we started, we didn't really have harmonized guidelines as such, but we wanted to start. So, what we decided to do was that, we're going to use the WHO pre-qualification guidelines in the interim, until we developed our own and adopted our own CTD guidelines for registration. Because we realized that, you know, if we waited for guidelines, etc, it would be a while before we started. Although in previous years, we had done some work on harmonizing our guidelines, these had fallen out of date, and they were totally out of kilter with what was going on with the WHO pre-qualification—and they were pre-CTD guidelines, so they really were no longer applicable. So, we just jumped in and started doing the work, and used the WHO guidelines. Within a year, we developed and adopted CTD guidelines in the SADC region. (Participant, SADC)

> At national level, we had to take into consideration the recommendations at regional level. So that for me worked well. And within that scope of development and review of guidelines. (Participant, SADC)
>
> What I would say for best practices, one is reliance, relying on work done elsewhere. Like we were relying on work already done by the WHO cooperative procedure. So, we did not need to do it again, we just adopted what had been done across countries in EAC. (Participant, EAC)
>
> I was in the initial inception of the East African Community medicines regulatory harmonization. So, I understand what did not work well in that area. But in IGAD, we've tried to design some regulatory documents that are very innovative and quite new. (Participant, IGAD)
>
> In 2017, we also adopted that policy for regional use, so that is what we call the ECOWAS harmonized common technical document. That's what is being used as the guidance for medicines registration. (Participant, ECOWAS)
>
> One of the things which we first did was to harmonize the documents, the guidelines, and the forms—the working tools that we use. And because of that, we were able to get best practices, the best practices in the world, because we were benchmarking on documents that have already been used by other more developed regulatory agencies. (Participant, IGAD)

However, other participants stressed that regional MRH initiatives have not been entirely effective, especially with regard to suboptimal/different legislation frameworks, administrative procedures, and technical guidelines.

> But also, the issues of having different legislation frameworks. And that's why we all want to, to ensure that we review our legislation to be in line with an AU model law. For us within SADC. I think we put those objectives very clearly at the beginning, so that we are also at the same level. (Participant, SADC)
>
> And sometimes to remember what type of information to share, again, is another issue. Which one is confidential, which one is not confidential…? that's another challenge. So, guidelines, policies need to be put in place so that we'll be clear what kind of information to share and how we should do it and on what regular basis. (Participant, EAC)

> There is good work which has been done on regional guidelines, but the implementation at the country level or tracking of implementation at the country level is often missing. So, it's not impossible to find one country with two guidelines, one regional and one national, and if these conflict, it's a big issue. (Participant, Industry)

.

A few participants, mostly from ECOWAS and the EAC, stressed that the MRH process has been a difficult process mainly due to conflicting interests from participating partner countries, as well as suppliers. They stressed that conflicting interests were mainly due to NMRAs' fear of losing funding and lack of a clear understanding of the whole MRH process.

> Some of the challenges with harmonization was the fact that people felt that there were those who would lose their funding, because a lot of regulatory agencies tend to be part of Ministries of Health. (Participant, ECOWAS)

> There were some technical guidelines that were a bit challenging to work on. Most of the countries adopted the guidelines from WHO, from the European Medicines Agency, and from the US Food and Drug Authority. So, you find they were having some differences, but through consultative process discussions, agreement, disagreement, you reach a consensus that you should include certain sections, or you should remove some. (Participant, EAC)

Work and information sharing

Almost all participants emphasized that work sharing has been possible due to harmonization of guidelines, countries within RECs working together, and sharing information. They stressed that teamwork has enabled participating countries to share what they are doing with more ease, helping both countries and RECs understand what others are doing and how they are doing it.

> What we did was that we had a whole working group that was for Information Management Systems [IMS]. They built the platform. And because they're from different countries, we put together all the different specifications. So, for every criteria or for every question or for everything that we needed to be in the management system, from the website, to frequently asked questions, to posting your data to, you know, maybe what our country was doing, we realized that this was a way that we could actually share information, apart from just putting information on product reviews. So that, in itself, having different countries come together to build a database, or to build a platform, I think was good, because they

brought in ideas from all the different countries, and we saw what worked. (Participant, ECOWAS)

We do have individual countries being quite advanced in their own information management systems. But it's now across the divide. So, the one sharing platform is actually MedNet. So, we keep all our dossiers or reports rather, on MedNet. And so, anyone who would require an assessment report, first of all, you go through the screening of having access to MedNet through your own agency, agreeing that you can be trusted to be able to download those. (Participant, SADC)

We’ve been together, we’ve been working as a team. At least now we could call each other anytime. It's quite different from the way it used to be before. We have our guidelines harmonized. So, whenever there is a product that needs to be assessed jointly, we have now the capacity, we have a pool of assessors already in our system, in everybody else's system. We have a pool of inspectors, we have competence assessment levels already, competence level one, two, and three. So, we have all these inspectors in those categories. So, it's easier to select. Depending on the type of facility to be inspected, you just choose the best inspectors you wish, depending on the dosage form as well. So that has been quite okay with us. (Participant, EAC)

One of the examples we identified as something that works well is in the EAC region, they're actually sharing the schedule of assessments. So that's really beneficial. (Participant, Industry)

I think most of the information was shared during the assessments and those meetings we discussed. But other platforms such as WhatsApp were established for inspectors and inspections, and information was shared. The most recent platform was formulated for substandard and falsified medicines. So, information regarding the regulators, meetings, reference documents, standard operating procedures, and so forth were shared on MedNet, which is accessible to country focal points. (Participant, SADC)

You may find that not all countries have the same expertise. Kenya has some strength, especially in pharmacovigilance and even registration of medicine. Our human resources are a bit more advanced, because of the institutions that produce these human resources. And with harmonization, we share this expertise. Because once an evaluation is done via what we call a common procedure, then we would adopt it in other countries, so they don't need their own. (Participant, EAC)

I suppose that's the only way of getting people together, to work together, the people with stronger capacity taking along or bringing up along the countries which have weaker capacity. That was one of the best practices, because during the harmonization processes, we set up technical committees for the different functions. These technical committees were based on experts in the region. We made sure we incorporated countries that didn't have experts but should be part of the process, even as observers. So, I think that was one of the best practices, for the stronger countries to bring up the weaker countries as a form of capacity building. (Participant, ECOWAS)

When it came to IT, the sharing of information, putting information in the website in IGAD, we decided the same. Ethiopia was agreeable, Kenya was agreeable, Uganda was agreeable, and there are products that are common across the countries. We just put them there. And they were available across IGAD. (Participant, IGAD)

Yes, the joint assessments have been going smoothly. Actually, in the joint assessment, every country is allocated a separate dossier and either the country becomes a primary assessor or second assessor depending on what you are allocated to. Normally either we become the primary assessor, or for some of the products, we are secondary assessors, so, we do assessments for medicines to determine whether they are eligible for registration or not. This is what we are learning from IGAD, so it increases our knowledge in terms of regulatory activities, including registration. This is how we are doing and how we are learning from our IGAD brothers. So it’s very helpful. (Participant, IGAD)

However, some participants, including those from industry, pointed out that work and information sharing is still challenging. This was attributed to lack of uniform information sharing platforms, the absence of an efficient set-up for applicants, and the failure of some member countries to enthusiastically engage in work sharing.

From the administrative side, also, having a single point of contact when it comes to the submission of dossiers and then efficient administrative activities and management is something that is useful to have. Sometimes country-specific requirements are not well clarified. (Participant, Industry)

It’s only on the IT component, sharing of information, it has been an issue for quite some time. And it has been quite tricky on our side to see how we could

communicate as we wish. But other objectives have been quite OK. (Participant, EAC)

And because everyone has got their own system, then you need this common platform whereby you can share information. So, this has been a daily challenge. (Participant, EAC)

And the information sharing, it hasn't materialized, we need to do more, keep sharing as we wish. (Participant, EAC)

I know WAHO has a website. And they tried, they tried to use that medium to share the information. But, you know, access was a little bit difficult. (Participant, ECOWAS)

The communication lag is too long. To me, I think that's the biggest challenge. (Participant, ECOWAS)

Information sharing is one of the complex areas. And this is because the countries like Tanzania, Kenya, and Uganda had already put in place information management systems, the systems they use for their processes for the activities. (Participant, EAC)

At the EAC level, we were supposed to have a portal to be able to access information that can be made public by all these other NMRAs and to be able to use the same to share our dossiers, our reports, and any other information in relation to safety, efficacy, and quality of medical products. So, we don't have a system in place at EAC currently. (Participant, EAC)

IGAD has an information sharing portal, it's called mrh.egad.int. But this sharing is more of information in terms of medicine data registered, but the problem that we have is management of that database, because most countries take time to upload. You have to actually tell them to upload, it’s not automatic. So, it’s not linked to what is being done at the NMRA. We tried to design it in such a way that it is going to be uploaded at the NMRA level, but it has not worked well. (Participant, IGAD)

The issue of information sharing is not good. No mechanism has been in place for information sharing. So, we can say that it's very weak. (Participant, IGAD)

> For EAC, the work-sharing system is not yet set up. So, we mainly send dossiers via Google Drive, sometimes the documents are too big… We need to build a work-sharing system for both regional blocs (Participant, IGAD).

> Sometimes there's a problem of work-sharing, because you have to ensure that one of the agencies that is more mature is grouped with the ones which still need help. (Participant, IGAD)

Training and capacity building

Most participants pointed out the importance of practical training, holistic training, and peer learning during the process of harmonization. Practical trainings helped NRMA staff learn hands-on skills for doing assessments, inspections, screening, and application reviews, among other tasks. In the harmonization process, participation in joint assessments has also helped enhance the capacity of member states.

> Interestingly, we actually developed the training module in-house for that. So, when people come into the agency, you train them first by learning how to do screening. And we learned that because of harmonization. So, you train people initially when they come in to be able to do screening, which is a basic part of any application review. And then, depending on how they progress, as capacity is built, then they become reviewers, etc. So, some of the best practices were learning how to do things together. (Participant, ECOWAS)

> Then another good practice that we adopted was that on the first and second day [of a joint assessment session], for 2 hours, we literally get someone to present on a topic, even if it's someone external. There's been times where we've actually even paid for an air ticket for someone, WHO experts, etc. So, each session incorporates a particular training. And those slides are actually then uploaded into MedNet, and then they are accessible to the different countries. (Participant, SADC)

> I have one brilliant example. I know we're probably not meant to say names. But Namibia was just the perfect example. They had almost literally no capacity. And now if I tell you that one of our best assessors is actually the head of evaluations in Namibia. It's that being so keen and learning from those around her, and then after a while, then they are also given a dossier. Then they will actually come and present until they are able to. (Participant, SADC)

There were capacity building trainings as part of our strategy for the harmonization. And we had training for all the areas, like for inspection, for registration, and also for quality management systems. So, we had regular trainings, which were facilitated by the lead, or the senior, experts from the five countries, and WHO was also participating as facilitators in these trainings. And actually, during the joint session, we used to have 1 or 2 days of capacity building by a senior expert from the Swiss Agency for Therapeutics, to train assessors on how to evaluate clinical data in the dossiers. (Participant, EAC)

I'll talk about Zambia. For us, for instance, conducting inspection of certain specialized facilities, like the biologic manufacturing facilities, which was a challenge before, but because of the trainings that were conducted jointly, we had competence on how to inspect such facilities, and also a number of dossier assessments of biosimilars, inhalers, for instance, labeling information, and also assessment of quality information, just to mention a few. And, as I've said before, WHO helped in organizing and ensuring that the needs that were identified by experienced assessors in the regions were addressed in those meetings. So, transfer of skill by competent officers in the region also was beneficial in terms of stability. (Participant, SADC)

We have had a number of attachments to other agencies, we have had experts coming over and training our people. We have had some staff going for some short trainings in universities, we have had some specialized training for our staff who are involved in quality control. And also, before the COVID lockdown, we had the junior GMP inspectors going for training in Ethiopia, and they were trained by WHO. So, it's a variety of training modes that are used for the training of staff. And then, the theory behind it is that when they return to their agencies, they're supposed to train their colleagues. (Participant, IGAD)

For IGAD’s trainings, it's not just about giving a lecture and going home. It’s not like that. For example, for registration, we are given some dossiers and we assess them in terms of checking the registration procedures. So, we have been trained in theory and practice. So, this is a hands-on training, not just the theory and go home, it is hands-on. Also, for GMP training, we have been taken to a facility in the pharmaceutical industry where we have inspected the premises, the staff, the equipment, we have checked what we have learned, we are taught in the theory. So, this is how they are doing it. (Participant, IGAD)

Much as participants expressed that regional MRH initiatives have contributed to capacity building, they also stressed that challenges still exist regarding the different maturity levels of various member countries.

> When you're doing assessments jointly, the levels of advancement are different. Sometimes there are a lot of exchanges, back and forth exchanges amongst ourselves, and sometimes that could prolong the process. (Participant, EAC)
>
> And for SADC, we have so many countries, so you can imagine being at different levels, but with very specific objectives. (Participant, SADC)
>
> We have new members coming in. When we were Kenya, Uganda, and Tanzania, we were moving very fast. It was easy to manage three countries. But then other countries came in: South Sudan, Rwanda, Burundi. They were at a different level of development. And Burundi and South Sudan were coming from very, very different political dispensations. (Participant, EAC)

Sustainable financing

Most MRH initiative participants noted that they have made some progress toward planning or implementing sustainable funding mechanisms to enable them to continue their work in the absence of outside support. For instance, ECOWAS now charges a screening fee for marketing authorization applications, which goes to the county that initially receives the application. In the EAC, the heads of regulatory agencies have adopted a Sustainability Plan, though it has not yet been implemented. SADC and IGAD have been implementing some activities on a cost-recovery basis.

> At ECOWAS, we are given a fee for screening. And it goes to the first country, which for us at this time is Ghana. It's just for the screening. And the rest is something that goes to ECOWAS, but we charge our own fee. Even through the process, you would still pay for the fees that you have to. And I think this promises sustainability. (Participant, ECOWAS)
>
> We have been conducting our joint inspections on a cost-recovery basis. So right from the get-go, our joint inspections have not been supported by partner funding. We have used the World Bank funding for capacity development for additional inspectors on the teams, who we feel we need to expose new inspectors to the process. Because we had already agreed ourselves that we're not going to use this as capacity development for new inspectors, we want to use it for actually doing work. (Participant, SADC)

> They get resources from what they are providing. For example, they charge for registration and GMP inspection. (Participant, IGAD)

> In EAC, the heads of agencies approved a sustainability plan. We are proposing a top-up fee to be charged over and above the national fees, so that if somebody wants to use this regional system, they pay a top-up fee, and then that top-up fee will be used to run the regional system. (Participant, EAC)

> Right from the beginning, what we had talked about was the fact that for those of us who are autonomous agencies, where we actually collect fees that should come in for running operations, if a product comes through the ZAZIBONA mechanism, of apportioning some of the application fees towards the initiative. Because we're looking ahead, we're saying donors are not going to be around forever. (Participant, SADC)

> Sustainability is one of the challenges. But at country level, like for Zambia, we've opened up new revenue streams, in terms of regulating what we call allied substances, which include medical devices, disinfectants, and cosmetics. (Participant, SADC)

Some participants from the EAC emphasized that it has a long way to go in order to reach a point at which external funds are no longer needed.

> The financial sustainability is also another thing which needs to be looked at, because you need money to push these things forward. (Participant, EAC)

Country ownership of regional harmonization programs

In all the regional blocks, heads of agencies reported a strong sense of ownership regarding the regional MRH initiatives. This ownership is demonstrated in several ways.

- Every quarter, the EAC convenes joint assessment sessions, and each country pays for its assessors to attend. These payments by individual countries have created a sense of responsibility and ownership. In addition, a WhatsApp group helps Heads of Agency stay in regular communication.

- In SADC, the responsibility for chairing the Heads of Agency meeting rotates among member countries; the country chairing is responsible for organizing the meeting. Responsibility for coordinating various activities also rotates. These practices have helped created ownership among the various Heads of Agency.

- At ECOWAS, constant meetings and open discussions take place among the different member countries. These discussions have helped create a sense of understanding and ownership.

The voices below describe ownership-building activities in more detail:

> We meet every 2 months to discuss—not applications *per se*—but to discuss challenges, we discuss what challenges you are facing. For example, I need support to put in a quality management system. How can we get the funding, or can another agency support? That was something that didn't exist before and only started with this harmonization. So, it shows we are happy with the process, we are looking even at a higher level to see how we can work together amongst us. I think ownership by just doing that in itself shows that yes, we have taken ownership of the process and we want it to succeed. (Participant, ECOWAS)
>
> I'll go the long-winded route but try and be very brief about it. The very story of ZAZIBONA, how ZAZIBONA got prominence, was through ownership. There was no money on the table and four heads of agencies said, right, let's get together and do something. So that was the first bit. And then having done that, they then actually moved to the next step. Then, at a time when the funding came on board, they had to decide on a model, to decide whether they are going to have maybe an office and people run it from there. But instead, the model that was chosen was to have a lead agency. (Participant, SADC)
>
> In SADC, you have medicines control authorities volunteering to do all the running around to coordinate and do this. So that, again, was leadership of one particular agency, but even prior to that, when there was no funding, they would actually rotate. So, you would be the coordinator for the first quarter or the first two quarters. And everybody took a chunk of the workload and the responsibility. (Participant, SADC)
>
> Political will is critical. Political will must be there. And it has to start from the top. As the head of agency, you need to show that you are interested, you need to show that with examples, that you are really keen on what everyone is doing. So, in our case, we have a WhatsApp group, everyone on one platform, Heads of Agencies, just to communicate on a regular basis on what needs to be done in our region, in our harmonization program. (Participant, EAC)

We rotated, since there were four of us, we rotated around each of the four countries to do that. And when we came to your country, you chaired the Heads of Agencies session, and you run the meeting, so that you also took ownership. So that it wasn't just the coordinating country, Zimbabwe, chairing everything, no, the host country did. So, we only chaired if we were hosting. I think that has helped a great deal. We've been involved right from the beginning. And that has actually created a real close-knit family of Heads of Agencies. Even when we have other issues that are outside assessments and things, we are always in touch. (Participant, SADC)

For some products in EAC, the countries have taken initiative even without funding from Bill and Melinda Gates, which gave us the initial funding. Right now, every quarter we do evaluation, especially of medicines of public health importance. And each country pays for the assessors to go for the common evaluation. And that is working well. Actually, it is to recognize that it is the same thing we are doing. We are not losing anything. But now we are doing it at a common level. (Participant, EAC)

I can tell you, for example, we have agreed Ethiopia becomes the lead country for medicines registration. And I can see from the three assessments and I can testify that the ownership by the Head of Medicine Registration in a particular country is very important in sustaining the activities. (Participant, IGAD)

For the EAC, for the last 3 years, we have adopted a cost-sharing mechanism. So how it works is the agencies host these sessions on a rotational basis. And then each country supports the experts to attend these meetings. And then the regional bloc, the EAC was paying for the air tickets, so a kind of cost-sharing. That's what we were doing before COVID, for all of 2019 that's what we did. And it worked. (Participant, EAC)

[Currently], we are working virtually, so the countries are supporting their experts to carry out this work. And they support them through provision of Internet services and time—the ownership is there. (Participant, IGAD)

Ensuring that recommended products are assessed and authorized within the agreed upon timelines

Since the beginning of the AMRH initiative, it has been a challenge to ensure that applications for joint assessment are reviewed promptly and that products that receive a joint assessment recommendation at the regional level are authorized at the country-level

within the agreed upon timeline. Most respondents reported at least slight improvement in this area.

> The countries have a 90-day window to allow certain country processes, etc. So, we are actually getting there. And since in this past year, 18 months, we've actually been able to now say that we are actively tracking and we're able to give particular figures for the indicator. (Participant, SADC)
>
> Initially it took some time to approve the product. But now, because they've been meeting many times and discussed this issue, because this is the cornerstone of harmonization, you need to speed up all the approval processes. But now at least the timelines are okay. (Participant, EAC)
>
> For EAC, timelines were a challenge in the beginning when we were going into the practical sessions of conducting joint assessment, sharing reports, and making regional recommendations. I think we have been making quite a lot of improvements. In terms of tracking timelines, we have come up with metric tools to measure the timelines from the submission up to the joint recommendation, and also from the joint recommendation to the market authorization at the national level. So, we have been tracking all the timelines for each product, and we have even a draft report related to the timelines for EAC. So, we are doing fine especially for registration. (Participant, EAC)
>
> I think for this year, national registration took between 31 to 30 days. There was quite a lot of improvement. And for the regional assessment of the older products… there are some manufacturers who have provided all the information, so you'll find that we don't have to ask for queries. So, you'll find that the regional timeline is between 0 to 180 days. (Participant, EAC)
>
> You know, when products are recommended during the assessment, individual countries could affect their decision at country level, following the in-country procedures. So maybe those in-country procedures would delay, and that's what we came to understand in one of our meetings, it’s again, the procedures, those are just recommendations. So, at country level, you have different procedures, but otherwise the timelines when we aggregate everything, I think the timelines were shorter, which was good. (Participant, SADC)
>
> The products that we are assessing, when all the manufacturer’s documents have been submitted and checked, if they are in compliance with the IGAD recommendation or IGAD procedure, then there is no need for further assessment.

> So, the best practice is that all these products have been imported into the IGAD countries without reinventing the wheel, without starting again the procedure. (Participant, IGAD)

However, the majority of respondents, including industry participants, stressed that adhering to the agreed upon timelines is still very much a work in progress. One particular problem emphasized was that even after a joint recommendation for a product to be approved at the regional level, individual countries continued to request additional information or impose additional requirements. In addition, the committee that approves new products in a given country may only meet a few times a year, which can slow approval at the country-level.

> There is room for improvement from that perspective. Because this process is still not quite clear. Even though in some of the regions there are procedures in place, usually timelines are not always followed. And also, there are some specific country requirements that follow this joint assessment decision. So actually, not just prolonging the timelines but adding to the complexity, because you've submitted a dossier, and then you need to submit additional documents or additional samples or additional whatever the further requirements are. (Participant, IFPMA)

> There are certain things that don't work so well, and they could actually be changed. Some of them is the pathways sometimes taking too long. The timelines are also not so reliable. What is written down is very attractive, but often we find that some of the pathways are taking much longer. (Participant, IFPMA)

> Yeah, that is still a challenge, because, you know, even when products have been recommended, there are still country-specific administrative measures that need to be fulfilled by the applicants. So usually we recommend, I think it is in our agreed timelines, I think it is 30 working days after recommendation. Sometimes this 30 day [timeline] is not possible because of the approval system in each country. So, what we are recommending is… it would be easier to issue marketing authorization certificates for each country if we had a synchronized, automated system. (Participant, IGAD)

> Well, I think the timeline. The process of getting other people to apply, doing the joint review, getting the results to the registration application process in-country. To me I think the timeline has been too long. (Participant, ECOWAS)

The other issue is that when getting the reports from different countries, I think we had a challenge. Others would take long to report back. For instance, if Zambia was responsible for an inspection report, I remember one of our officers being really cautious that they needed to finalize and submit inspection reports in a timely manner. So sometimes it's the delays by the technical staff to bring back the report after an inspection is done or assessment of a dossier is done. So those delays can again cause you to do the work quickly. But then writing the inspection report can take some time. (Participant, SADC)

It's the countries that are delaying. There is the regional economic bloc and there is the national regulatory authority. Those two are different. So, the marketing authorization or registration is offered by the national agencies, not by the regional economic blocs, so there's always a delay after a regional decision. (Participant, IGAD)

Consistency of guidelines at the country level

One industry participant voiced concern that, with the advent of the regional MRH initiatives, some countries have multiple, inconsistent sets of guidelines, making marketing authorization assessments and GMP inspections unnecessarily confusing and frustrating for applicants.

There is good work which has been done on regional guidelines, but the implementation at the country level or tracking of implementation at the country level is often missing. So, it's not impossible to find one country with two guidelines, one regional and one national, and if these conflicts, it's a big issue. (Participant, IFPMA Industry)

OBJECTIVE 2: TO EXPLORE THEMES SUCH AS PRACTICES THAT DID NOT WORK WELL, THE INDUSTRY EXPERIENCE, BEST PRACTICES, CHALLENGES, AND FUTURE DIRECTIONS

Practices that did not work well

Respondents identified practices that did not work well as reinventing guidelines unnecessarily, getting bogged down by trying to establish novel information sharing

platforms, making unnecessary queries during the joint assessment process, and creating centers of power in one country.

> If you want to harmonize the quality management system, the ISO standards, all those, it's not easy. The good thing is just to start with the simple items. Harmonization of guidelines, this is straightforward—you're not reinventing the wheel, coming up with your own guidelines. No, you can just adopt international guidelines. (Participant, EAC)

> Information sharing, I wouldn't wish for them to begin early. They could do it maybe at a later stage because of the challenges I've just mentioned. But, of course, now It all depends on how do you get on with each other. (Participant, EAC)

> As far as things that we would encourage the initiatives to avoid, one of them is so-called unnecessary queries between Ministry of Health and reviewers. I think there should be, and this is part of capacity building, the expected kind of queries that you could get once a submission is made. Then more importantly, is additional dossier submissions to each NMRA. So, if you have an application being made, and it is assessed positively, there should be room for the NMRAs not to ask for you to, again, make a fresh application. Of course, it's usually the same dossier submitted to each of the NMRAs. There is a certain level of duplication which we see there. (Participant, IFPMA Industry)

> Then the other one is, they should not try and create centers of power in one country. They should not, for example, make one agency become the super-regulator in the home because it brings political issues, they should try and distribute the workload among different RECs, at different countries, so that at least there is capacity. (Participant, IGAD)

Openness of regional initiatives to change, and learning that occurred between initiatives

Almost all respondents reported that the regional MRH initiatives are open to change and have been learning from one another, as well as from regulatory bodies abroad.

> But the one thing that we quickly picked up was the issue of wanting to get our guidelines sorted. We approached EAC and asked if they could actually assist with whatever they have. And then we looked at whatever was in their compendium and then tried to build up from that. (Participant, SADC)

> Well, the other thing that we adopted is not necessarily from a region, but more from the WHO, the issue of reliance. So, when it came to biosimilars, the original approach was, we first said, “No, we don't have the capacity.” But then we also started grooming capacity at the individual national level. So, it's only now that we have a guideline. But before that, we actually had a lot of input from the European Medicines Agency. (Participant, SADC)

> We are trying to establish now our regulatory system. For us, the AMRH program is very helpful, because we will just adopt what we have learned from our predecessor regulatory authorities. (Participant, IGAD)

> The EAC is taking the lead. I think most people are learning from us. We learned from initiatives, like from the European Union, that were good. When it comes to pooled procurement, we've seen something from Asian countries and the Gulf States. We thought it was good, but we are not yet there. (Participant, EAC)

Whether the process of assessment and inspection has gotten easier for industry as a result of regional MRH initiatives

Industry respondents reported that the movement toward regional MRH is encouraging. In particular, participants were pleased that the harmonization process had resulted in formal guidelines and processes and greater professionalism, and that, theoretically at least, joint assessments and inspections could result in time and resource savings for applicants.

> Overall, this has been a positive development in terms of having the process of assessment and inspections. And one of the things, of course, would be actually positive guideline development, that guidelines which never used to exist now are there in one way or another. It's a question of whether they have been fully implemented and to what extent they are being implemented. That is something else. But the guidelines are there. (Participant, IFPMA)

> Some of the manufacturers that participated in some of the joint assessment procedures and also joint inspections, we noticed that it is, in general, beneficial to join those pathways, because we see overall time saving and then, in some cases, also procedures that are more transparent and more predictable compared to the national ones. And what we also see as a benefit is that the timelines are shortened through the regional joint assessment procedures compared to national. (Participant, IFPMA)

> Once you have the guidelines, there is a certain level of accountability that comes with it. Because these are points of reference, unlike in the past, where some countries, of course, don't have the guidelines. There is also to a certain extent, an increase in capacity for the countries, because as guidelines now are becoming available, some of the countries which were really not having a lot in place, they've vowed to catch up in a positive way and also to learn from their peers. So, peer-to-peer learning has also been there. (Participant, IFPMA)

> The other one is for ZAZIBONA, where the so-called module 225 of the dossier is actually reviewed together by the member countries. And this, what I would call here peer review, really helps also build capacity for the assessors. Also, on a positive note, especially for ZAZIBONA, is that the certain level of agility by the assessors when they are following the set guidelines, ICH standards and the rest, when the requirement in the regional guidance is not fully applicable for the product assessed. So, there is a certain level of giving life to the guideline, which also makes things a little bit more practical there. (Participant, IFPMA)

However, a participant from a local generics manufacturer reported that, in practice, the regional MRH initiatives were not living up to their promise as engines of greater efficiency.

> The harmonization initiatives have not really made anything easier for local manufacturers. Maybe for international companies. (Participant, Industry)

How industry has experienced regional MRH initiatives' joint assessments and inspections

One industry participant reported positive experiences with joint assessments and inspections, though it should be noted that these processes, especially inspections, have been disrupted by the COVID19 pandemic.

> For some of the manufacturers that participated in some of the joint assessment procedures and also joint inspections, we noticed that it is, in general, beneficial to join those pathways, because we see overall time saving and then, in some cases, also procedures that are more transparent and more predictable compared to the national ones. And what we also see as a benefit is that the timelines are shortened through the regional joint assessment procedures compared to national. (Participant, IFPMA)

However, another participant, representing a local generics company, reported a less positive experience. To date, his company had submitted nearly 20 applications for joint assessment, and he reported roughly half were stuck in limbo.

> My company has gotten two products approved through the regional processes but has not found it to be an easier process than applying to individual countries. (Participant, Industry)

Willingness of industry to pay higher fees for joint assessments and inspections than for individual country assessments and inspections

Most industry participants reported that they would be willing to pay higher fees for joint assessments and inspections, if these regional processes ensured predictable and efficient timelines. However, they voiced concern that, at present, joint processes were not necessarily delivering greater efficiency and reliability.

> The issue of the fees needs to be looked at in a balanced way. There is a need to make the process sustainable, because this is another layer of process compared to the national one, to a certain extent there is some level of coordination. On the contrary, the fees, even as it is done nationally, need somehow to be justified. (Participant, IFPMA)

> If you look at it from the perspective of industry, this is not a simplistic additional fee between the member countries, and what they normally charge. There needs to be a little bit more metrics built into that. Specifically, I think there needs to be an exchange, for instance, from industry, thinking about “Is there a transparent fee system?” It's not just to increase the fee or reduce the fee, but what is the system in place? So, it's not easily a question of yes or no, but it's a question of, under what circumstances can the fees be increased or reduced? (Participant, IFPMA)

> One other thing, which is a big ask also from the industry, would be predictable timelines, and also reliable processes and timelines. You know, more like a guaranteed level of service that applicants could get. So, if you use that as a way to make the argument, then it could also mean that this system could actually lead to maybe a little bit of increase of the fee, but with no counterbalance by something solid, not being given back. The current system doesn't necessarily guarantee outcome. And this kind of guaranteed outcome is what there needs to be, watertight. That you pay this kind of fee, or it could even be, let's say graduated, it could potentially also have something like a Fast-Track kind of thing, depending on the kind of product which is being applied for. But in the end, these deliverables need to be there. (Participant, IFPMA)

> It could be that there be a mechanism of compensation for NMRAs which perform the dossier assessment. I think there is such a model in Europe, the so-called European Medicines Agency model, as this can be used to support the joint assessments, which is needed from the NMRAs. Because the NMRAs are using their resources to send the people to make the assessment. (Participant, IFPMA).

> If it is less than a 10% increase, probably no one will object. But that's if a benefit is provided in terms of timelines. (Participant, Industry)

Whether industry feels its feedback has been included when regional MRH initiatives create plans or develop guidelines

Industry participants voiced their desire to be included in the work of regional MRH initiatives, especially in planning and developing guidelines.

> I think we have seen good things coming out of the different regions. And we could see that because IFPMA, since the start of the AMRH initiative, was part of the discussions at the steering committee level and is a partner of the initiative. Through this, there is continuous engagement with the key players of the different regional Secretariats. So, we would take any opportunities, face-to-face meetings, when we would have the steering committee meetings, but also sometimes the scientific conference organized by NEPAD to ensure that we would meet all these regional Secretariat representatives, discuss our challenges, discuss our successes, and try to also see where we can together improve, and harmonization of our work in the regulatory fields. (Participant, IFPMA)

> Another important point would be related to the benefits that the regional procedure brings versus the national procedure. So, we are willing to work with all the stakeholders to actually address this challenge and to make sure that, you know, all the concerns from different sides are really addressed. (Participant, Industry)

However, industry participants did not feel that the regional MRH initiatives necessarily valued their feedback or made it easy for them to participate in planning or feedback sessions.

> Also, sometimes the initiative already has their agenda, and inviting industry to participate is really just rubberstamping. Their feedback isn't really wanted. For example: East African manufacturers had proposed that they would be given a

certain preference compared to foreign manufacturers, but EAC wasn't interested, wouldn't listen. (Participant, Industry)

But this is also where we see the challenge that we are not so well informed of what is in the pipelines for these different regional Secretariats. And we would really recommend that they could share maybe in advance a work plan for the coming years. Now I don't know what would be the vision for the coming years, because we all have now as a target AMA. And how these regional guidelines would converge to harmonized continental guidelines is another question. But I think sometimes when we share our comments it is through our trade associations, but we don't know at the end, for example, the process—and the process is not so transparent. We don't know how the feedback is used, or how industry could better support, at the end, this regional initiative going forward. (Participant, IFPMA)

The issue with the regional guidelines, when we were informed of these guidelines, sometimes late, we would have maybe 2 weeks to react. And even sometimes we knew that, for example, the OCEAC variation guidelines, we knew that they would be coming, we knew that they would be having a consultant working on them. But the contract with the consultant was so short that at the end, no time was left for stakeholders to provide comments. And even if we could provide comments, then the comments were not maybe considered, because they really wanted to wrap up the project, which is not a good practice. (Participant, IFPMA)

The biggest challenges industry has faced in adapting to changes made by the regional MRH initiatives

In general, industry participants had a number of suggestions about how regional MRH initiatives could improve their interactions with industry.

I think the lack of awareness of the processes, for the processes themselves, but also on the benefits for all—that can be improved. (Participant, IFPMA)

The unpredictable timelines, in terms of planning of the joint assessment, at different levels, timelines for dossier submissions, timelines for dossier review, timelines for queries, and timelines to get back to manufacturers on the queries or timelines for the end, receiving the approval and the authorization at national level, can be done a bit better. (Participant, IFPMA)

I think one point we also want to highlight is that the scope of the joint assessments, the list of products, is too restrictive. And that's been an issue, we have seen it for example, in the ECOWAS joint assessment process. There should be a

mechanism to include products of interest and also allow innovative medicines rather than just old products to be in the pipeline. (Participant, IFPMA)

The lack of transparency in the assessment calendar [is a problem], I think this has already been alluded to, and upcoming milestones, how many products are in the pipeline, what is the kind of workload which is there, when is the next assessment time? (Participant, IFPMA)

Trust between regulators is a big challenge. (Participant, Industry)

For manufacturers, there are still many commercial barriers, tariffs, etc. that make things hard. (Participant, Industry)

Changes that industry would like to see the regional initiatives make

Industry participants also gave specific feedback about changes they would like to see MRH initiatives make in the future.

Regulators and donors should focus on local manufacturing by giving waivers to local companies, holding their hands to get through the quality system. (Participant, IFPMA)

Another point that will be very useful is developing trust between NMRAs that are supposed to collaborate. That would enhance their collaboration, and that would support them to eventually really rely on the work of the others. (Participant, IFPMA)

Relatedly that is linked to the above issue of trust building among national regulators, is making sure that the regional procedures are adopted at national level, and keep them somehow complimentary and not competitive, making sure that regulators see the value in participation in the regional procedures, and not as a threat to their national regulations and processes. Adherence to timelines, and not just the timelines, but in general, if there are procedures in place, then those procedures should be followed through as much as possible. (Participant, IFPMA)

Definitely the WHO guidelines and public consultation process is something that we would recommend. And also, we'd like to encourage the RECs to develop a sort of work plan for a couple of years or 5 years, not only for new guidelines, but also for any guidance that would be getting old and need to be revised. And also maybe a clear indication and more transparency on the regional website

> Secretariat when they exist, of what is the status of the guideline in terms of implementation, whether it's adopted, whether it's implemented in all countries in the region—this would be really helpful. (Participant, IFPMA)

Key Best Practices for MRH Initiatives

“Twinning” between more and less mature regulatory authorities

Multiple participants mentioned “twinning” as a best practice for capacity building within regional MRH initiatives. This strategy helped improve the regulatory capacity of less mature NMRAs, building trust within the initiatives.

> Facilitating twinning between member states: I think that was important, because one country would go to another. Say, for instance, if you just see that one country is better in inspection, it was good that you go to that country and learn from them. For instance, Zambia, for us, in terms of quality management system, that's important, because you need to have procedures and so forth standardized, so that you are all speaking the same thing, there's consistency, there is accountability and transparency. (Participant, SADC)

> In 2015, we began what we call mentoring and twinning, where the countries visit, spend a month. They moved across the region to spend some time, 2 weeks, 3 weeks, or a month with each other, the French moving to the English, the English moving to the French. They just look at what each other is doing, learn from them, build that trust and confidence. So, it's was a time for them to understand each other and to work as a region and integrate themselves. So that helped. (Participant, ECOWAS)

Joint assessments and inspections

Several best practices that facilitated work sharing were highlighted by participants, including performing thorough screenings in advance of joint assessment sessions, involving assessors and inspectors from multiple countries in each joint activity, capitalizing on individual member countries' strengths, publicizing the schedule of joint assessment sessions ahead of time, and asking all marketing authorization applicants to sign a consent form to undergo joint assessment.

> What happened was that for joint reviews, which is what we call working together to evaluate an application, what we did was we selected three countries. We had a screening process, and then the review process, and then the approval process. With the screening process, within our region anyway, and I think it's the same with

the other regions, we select the three countries that will do the review. For us, it was Ghana, Nigeria, and Burkina Faso—a mix of languages, a mix of capacity. And we did the initial screening. Once the screening was done, you know, the applicant had submitted and the screening was done, all the questions that needed to be asked and making sure the application was full, was done. And in that process, even by itself, we had to, for us, and for most countries, set up systems that maybe we didn't have. In a lot of countries, applications come in and are immediately processed. But that is something that we all learned that, look, it is possible to do a screening. And Canada was a country that we remember did screening, but we never actually did it. (Participant, ECOWAS)

I think it's the openness that was there in terms of when you discuss, and you have your reports, and there was a second, that one report was peer reviewed by the second country. So, in that way, it wasn't just one country giving all the information. There was also a second country that also reviewed their reports, so there was a lot of openness and ensuring that they learn from one another. (Participant, SADC)

Actually, in the joint assessment, every country is allocated a separate dossier and either the country becomes a primary assessor or second assessor. So, depending upon what you are allocated to. Normally either we become the primary assessor, or for some of the products, we are secondary assessors, so, we do assessments for medicines to determine whether they are eligible for registration or not. (Participant, IGAD)

The collaboration between the regulatory authorities has helped each country to learn the areas of competence of different regulatory authorities and also identify the more skilled regulators for different specific areas. I will also use the same example of Rwanda FDA, because it was established in 2017. They've been able to work with the Tanzania Medicines and Medical Devices Authority and also the National Drug Authority of Kenya and the Pharmacy and Poisons Board by engaging the experts from these advanced regulatory authorities to go and train their personnel in different areas, from inspection to registration, quality control, quality management systems, evaluation of dossiers, and how to inspect a facility. (Participant, EAC)

Well, I'll say we were borrowing the strength from other countries. Like Uganda was a bit more advanced in GMP inspections, because they started much earlier. And well, we would adopt reports from that country, the ones they did. What they would do was common, in a spare class when all the countries would be present. (Participant, EAC)

> One of the examples we identified as something that works well is in the EAC region, they're actually sharing the schedule of assessments. So that's really beneficial. So, you could plan better your application and also the resources needed to support that application. So, if there is a possibility to establish similar practices, that would be really useful. So early enough and widely enough with the applicants. (Participant, IFPMA Industry)

> I've told you about the consent form, which is not [used] in the EAC. I have told you about the domestication tool SOP, let me call it standard operating procedure (Participant, IGAD)

Expert working groups

Convening expert working groups for various aspects of the regional MRH initiatives' work was also mentioned by many respondents as a best practice. These groups not only helped the initiatives move forward in different areas, they served as a mechanism for the various countries to get to know one another—and for less mature NMRAs to improve their regulatory capacity and take leadership roles within the initiatives.

> The fact is that we broke into the different working groups. We had the registration working group, the clinical trials working group, the information management systems working group, and others. Once we were in our different working groups, and each working group was constituted by members of the different countries within ECOWAS, what we did was we looked at our strengths. So, you joined the working group, depending on the strength of the country, which was best. And then as well, we also had some people who actually also were made to join working groups, because maybe their capacity was slightly lower. (Participant, ECOWAS)

> In 2015, the heads of NMRAs came together to form the West African Medicines Regulatory Harmonization Steering Committee, from which seven expert working groups were also created. So we had the registration group. We had the GMP inspection, QMS [quality management system], the IMS [information management system], the policy and legislation group. In all, seven groups were created. And each of them have what is called their terms of reference, working guidance. In 2018 March, they were officially integrated. That was done under the projects we had support for in the form of the trust fund from the World Bank, which is the AMRH Consortium. That support was one of the crucial elements, the key instruments supporting this part. (Participant, ECOWAS)

Benchmarking as a way to build trust and improve regulatory capacity

Multiple participants mentioned that having member countries participate in WHO's global benchmarking assessment process was a key way to objectively build trust and improve regulatory capacity. Organizing member countries to complete this comprehensive assessment was not always easy, but regional MRH initiatives reported that it was worthwhile.

> For SADC, we have so many countries, so you can imagine being at different levels… For those that had a certain [WHO] maturity level, we wanted them to progress from either two to three, and those that were at the lower level to progress to another level. I think, what helped was also the benchmarking, self-benchmarking, so that you know at what level you are, and then you also develop the institutional development plans, which we are now following up the implementation of those institutional development plans that were done at country level. So that has helped. (Participant, SADC)
>
> When the global benchmarking was done, two countries missed the [benchmarking] exercise because of communication challenges with WHO. And WHO couldn't go back and do it with them. So then they asked the lead agency to say, "Are you prepared to do this on our behalf?" And because we had the funding from World Bank, we asked if this could be included now as an activity, and they said, they were happy to support it. And so when the contingent went to train them on that, the Head of Agency also went for a day and a bit. And that made a huge difference, because she met with all the top people there and got to explain exactly what it is we're about, what they stand to gain, and you know, that they we don't want them being left behind. And voila, we are seeing a lot of participation from them now. (Participant, SADC)

Challenges Faced by the Regional MRH Initiatives

Respondents reported numerous challenges faced by the regional MRH initiatives. Here we include insights into some of the difficulties most frequently cited.

Lack of resources and time

Participants shared that one of the most persistent challenges encountered by regional MRH initiatives was insufficient funding, time, and resources.

> We had a few logistical challenges. Things like nominations. It was a marathon 18 months [for which SADC activities received external funding]. This was the shortest

18 months of my life. It's a good lesson learned. We had this very ambitious work plan, which had a lot of activities. And one of the things I was recording, like we're doing the project closing report, is that sometimes we probably needed to look at high-level impact and maybe less in terms of the number of activities, especially if you know your implementation time is a bit reduced. So logistically, you had to send out invitations, and say, give me your nominees. Within so many days, we needed to book flights, etc. And sometimes we would have like three, four different activities going on, in different countries at the same time. (Participant, SADC)

The initiatives require strong financial support. This has been a challenge in having sufficient numbers to even attend meetings and so forth. In my view, financial support, we need that. Without that, really you cannot. (Participant, SADC)

[With regard to challenges,] I think mostly lack of funding. (Participant, IGAD)

Staff turnover

Participants also cited high levels of staff turnover at NMRAs as a formidable challenge, as such changes affect the level of support for regional MRH initiatives and also the availability of the expertise needed to keep the initiatives functioning smoothly.

The heads of agencies are changing, and new ones are coming. And depending on the attitude and all that, sometimes you need to engage people and show them why this thing is really important, you need some time to convince them and get them on board to be engaged. (Participant, EAC)

Besides lack of funds, there are a lot of people who are not aware that there's a lot of high turnover. The region has a lot of turnover in terms of staffing, especially on Heads of Agencies, it has not been very comfortable. Especially we've had a lot of changes in Uganda, changes in Sudan, changes in Somalia. So, it has really affected the implementation in terms of the governance structure. (Participant, IGAD)

Language barriers

Participants from ECOWAS reported that the numerous languages spoken with the region (including English, French, and Portuguese) had posed problems, especially early in the initiative's history.

It was the language barrier, because we had the Arab Maghreb Union and we had ECOWAS. So, we had one group going in one direction and another group going

> in another direction. So language was seemingly our biggest barrier (Participant, ECOWAS).
>
> It took ECOWAS a long time to start harmonization, basically because of an interesting language complication. Well, we have English and local Francophone and Lusophone. (Participant, ECOWAS)
>
> [Speaking about challenges faced by other regional MRH initiatives] One thing that I've learned is language. You have French-speaking and English-speaking countries. Sometimes there's some kind of heated exchanges, and agreeing on some items is a bit of a challenge. (Participant, EAC)

Information sharing

The lack of a common information sharing system was mentioned over and over again by participants as a major challenge faced by regional MRH initiatives. Some initiatives reported trying to use MedNet for various work-sharing functions, rather than building a new system from scratch, but this did not work well for all purposes.

> Dossiers are confidential documents of manufacturers, and they need to be handled carefully so they don't spill out to the public. We've been trying to use WHO's MedNet server. But that server has issues, so it has not been good. So, we still have a lot of challenges and sharing of dossiers. That's a big challenge that most RECs have encountered. (Participant, IGAD)
>
> NEPAD is doing one for the continent. We have been doing one for SADC. And the reason why we want to do one for SADC is that we feel that the MedNet system is a little restrictive. You can't manipulate the information once it's in the system. You can only place it in the system, and people can have access to it. But you can't manipulate it, you can't do much with it. We would like to have a system which would be able to give us statistics that can track where applications are, etc. So that's why we are trying to develop our own system for the region that will allow the flexibility to track. (Participant, SADC)

Lack of transparency in the assessment calendar

Industry participants mentioned multiple times that the lack of transparency in regional MRH initiatives' assessment calendars was a major problem for applicants. Here, SADC's regular and publicly available schedule was cited as a best practice. Participants from the regional initiatives themselves seemed less concerned about regular and transparent assessment schedules.

> The lack of transparency in the assessment calendar is a big concern and upcoming milestones, how many products are in the pipeline, what is the kind of workload which is there, when is the next assessment time. (Participant, IFPMA Industry)

Future Prospects for Regional MRH Initiatives

When it came to the future, participants had many ideas about beneficial changes. In addition, many reported optimism about regional and continent-wide harmonization initiatives, provided sustainable funding could be secured.

Developing functional information sharing systems

No regional MRH has a functional information sharing system set up that is common across all member countries. However, the importance of having such a system was mentioned over and over. Participants emphasized that they wanted their systems to be comprehensive, including functions above and beyond the core information needed to conduct joint activities. Desired functions included e-learning, quality management, competency building, and much more.

> We are also creating what we call the web portal. There'll be a SharePoint. That portal has so many areas of the regulatory functions. And there is SharePoint that, when it's complete, you can get communication, e-learning, and so many aspects. (Participant, ECOWAS)

> Initiating the competency framework has also been built up a lot in terms of how we will be looking forward to, so that's another thing that we will be pushing ahead, to say that how do we now build a database. And what will that database have? So, it will speak to competency, it will speak to training needs, it will also speak to just the normal day-to-day activities, like joint assessments and joint inspections, and then have a pool of people that you can actually now call. (Participant, SADC)

> One thing that we're now going to spearhead is that we do want a quality management system within our setup. (Participant, SADC)

Capacity building

Participants mentioned the need to continue working at building capacity, to foster trust between member states and also to facilitate the ability to scale up joint activities.

> Where are we in terms of capacity? We're trying to design a new training program with them where it is a practical training, not theoretical. You know, the problem is most of these trainings and workshops are too theoretical to offer the best advice. (Participant, ECOWAS)

Funding

Recognizing the importance of funding, participants described their plans to attract support in the coming years. Whereas newer initiatives were still seeking external funding to establish themselves, more mature initiatives were seeking to establish self-sustaining funding mechanisms.

> Yeah, we are still discussing with the World Bank to fund IGAD MRH. It's something that is under discussion now, that is going to be considered in that funding. (Participant, IGAD)

> In future, we're looking at having a coordination fee. So that if [applicants] say they want a joint assessment, and they want us to convene a meeting, we can be able to convene say five meetings in a year. (Participant, IGAD)

> In some instances, countries may have to chip in for it to be a success, or ECOWAS also to be supported through the country contributions. But sustainable funding is usually a problem, especially when we're thinking that countries should come together, do joint reviews. (Participant, ECOWAS)

> In the long term, we really ought to put in the mechanisms at regional level. It means writing these complicated documents, unfortunately, that are way beyond me, that is at secretariat level, where governments sign up to make sure that they support this initiative. Because it cannot just rely on willing heads of agencies. It has to be at government level. (Participant, SADC)

Expansion to new areas

Some of the more mature regional MRH initiatives plan to expand their activities to encompass new areas, such as active pharmaceutical ingredient (API) manufacturing. Industry participants also voiced interest in the initiatives expanding their regulatory activities.

> We will need to create some sort of certification process for API manufacturers. So that once they are certified, and they come and apply for the finished pharmaceutical product, we don't need to go now into the evaluation of the API

data, because we have already done that. It is a procedure that is not being used or applied in any NMRA… And also maybe receiving other categories of drug products, like more biotherapeutics and other complex molecules. (Participant, EAC)

Additionally, joint assessment procedures for the initial registration. And then afterwards, that's not the end of the product lifecycle. That's just the beginning. So, thinking about a holistic approach to the whole lifecycle of the product from this joint assessment perspective would be really useful. Because for the time being, we have regions assessing initial marketing authorization, and then there are different national requirements for the post-approval changes. And eventually, we're having quite non-harmonized dossiers in different countries. So, if there could be opportunity to harmonize also variation-handling processes and requirements that would be useful. (Participant, IFPMA)

Word frequency cloud

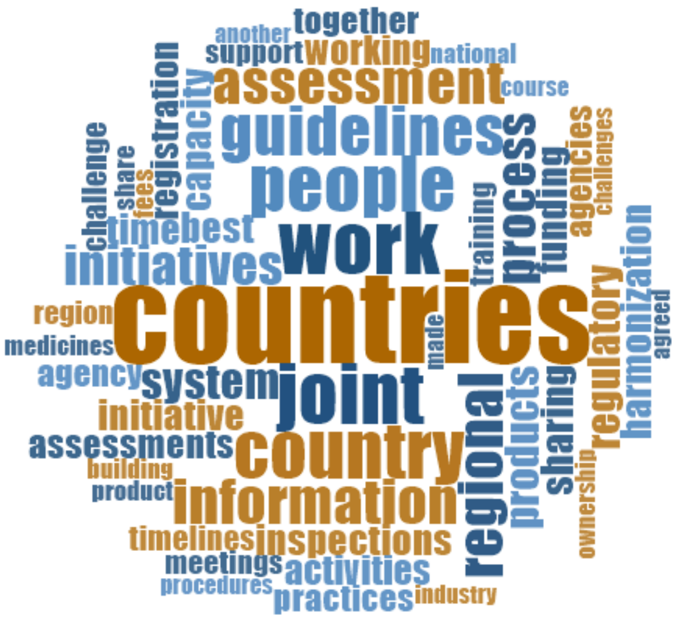

Supplement: S3 Text — (PDF) [file pgph.0001651.s003.pdf]
